# Supplementary material for: Improving deep models of protein-coding potential with a Fourier-transform architecture and machine translation task
Source: PLoS Comput Biol. 2023 Oct 12;19(10):e1011526. doi: 10.1371/journal.pcbi.1011526 (PMC10597526; doi:10.1371/journal.pcbi.1011526)
Supplement: S1 Text — Additional methodological details and results for hyperparameter tuning, training, and interpretation of models. Includes supplementary tables and figures as referenced throughout. (PDF) [file pcbi.1011526.s001.pdf]

Supplementary Methods : Improving deep models of protein-coding potential with a Fourier-transform architecture and machine translation task

Joseph D. Valencia[1], David A. Hendrix[1,2,*]

October 4, 2023

1 School of Electrical Engineering and Computer Science, Oregon State University, Corvallis, Oregon , United States of America

2 Department of Biochemistry and Biophysics, Oregon State University, Corvallis, Oregon, United States of America

* Corresponding author: david.hendrix@oregonstate.edu

A Architecture and training details

The short-time fast Fourier transform in LocalFilterNetwork was implemented with the torchaudio library [1] using the Spectrogram and InverseSpectrogram classes. We used pad_mode='constant' to pad the input with zeros where the LFNet window overhangs the sequence, which has the effect of upsampling the sequence within such windows to match the filter frequency resolution. We also padded batches to accommodate input examples with different numbers of tokens.

We implemented hyperparameter tuning with the Ray Tune PyTorch library [2] and the Bayesian Optimization HyperBand (BOHB) method [3]. We chose BOHB because it allows models based on different hyperparameter settings to be evaluated simultaneously while terminating less promising trials early for a desirable degree of parallelism and efficiency. At the same time, BOHB uses Bayesian

optimization to adaptively sample from the hyperparameter search space for improved model performance. One of the four initial settings for seq (LFN) and class (LFN) was set to dim=64, #enc=12, #dec=12, dropout=0.2, lr warmup=4000, LFNet window=200, and LFNet L1 sparsity=0.5, as this was found to be a good starting point during model development. The range of hyperparameters that we allowed to be randomly sampled is shown in Table A and Table B lists the optimal set of hyperparameters found for each model type. We used the learning-rate warmup schedule from [4], with the number of warmup steps as a hyperparameter. The λ value for seq-wt was determined via a small sweep on top of the best hyperparameter settings for the unweighted bioseq2seq LFN and CNN because an initial evaluation found that including λ in a full retune harmed performance.

Range
Model dim = [32,64,128]
Encoder layers = [1,2,4,8,12,16]
Decoder layers = [1,2,4,8,12,16]
Dropout probability = [0.1,0.2,0.3,0.4,0.5]
Learning rate warmup steps = [2k,4k,6k,8k,10k]
LFNet window size = [100,150,200,250,300,350,400]
LFNet L1 sparsity multiplier $\sim LogUniform$(1e-1,1.0)
CNN kernel size = [3,6,9]
CNN dilation factor = [1,2]
Positional decay rate = [0.01,0.05,0.1,0.5]

Table A. Hyperparameter search space

Model	dim	# enc	# dec	dropout	lr warmup	LFN window	LFN sparsity	seq-wt λ	CNN kernel	CNN dilation
seq (LFN)	64	12	2	0.2	2000	250	0.004	0.1	X	X
seq (CNN)	128	8	2	0.5	2000	X	X	0.05	6	2
start (LFN)	64	16	X*	0.1	4000	200	0.001	X	X	X
start (CNN)	64	12	X*	0.2	4000	X	X	X	3	2
class (LFN)	128	16	16	0.1	4000	200	0.011	X	X	X
class (CNN)	64	4	12	0.4	8000	X	X	X	9	2

Table B. Optimal hyperparameters found during tuning by training task and architecture. The bioseq2seq and bioseq2seq-wt differ only by the addition of the λ value for rescaling the loss function. *The decoder is fixed at a single Pointer Network for seq2start.

B Extended prediction results

The LFNet filters learned different frequency-domain strategies in the two model types, with bioseq2seq-wt (LFN) weights having more extreme phase values, as seen in Fig D. Phase values for the 3-nt band are shown as a kernel density plot with the remaining periods as a histogram, with bioseq2seq-wt

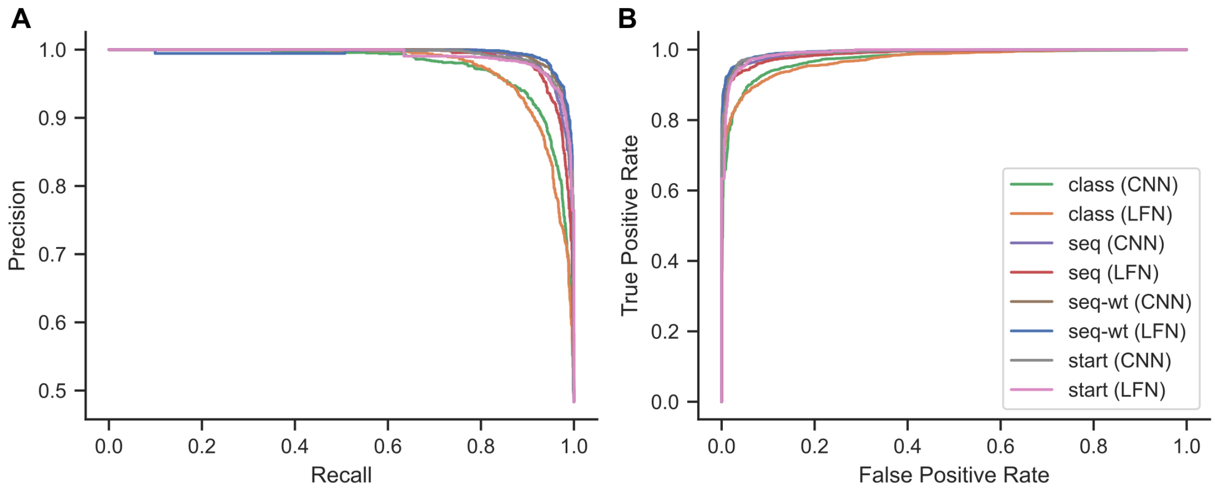

Figure A. Threshold-independent classification performance. (A) Precision-recall curve (B) Receiver operating curve

model	F1	recall	precision	MCC	AUROC	AUPRC
CPAT	0.938	0.950	0.927	0.880	0.985	0.987
CPC2	0.911	0.859	0.970	0.843	0.981	0.982
RNAsamba	0.956 ± 0.001	0.947 ± 0.002	0.964 ± 0.002	0.915 ± 0.002	0.991 ± 0.001	0.992 ± 0.000
class (CNN)	0.919 ± 0.006	0.900 ± 0.018	0.938 ± 0.012	0.846 ± 0.011	0.976 ± 0.003	0.977 ± 0.003
class (LFN)	0.911 ± 0.003	0.896 ± 0.012	0.928 ± 0.015	0.832 ± 0.008	0.974 ± 0.003	0.976 ± 0.002
seq (CNN)	0.955 ± 0.004	0.954 ± 0.007	0.956 ± 0.012	0.913 ± 0.008	0.992 ± 0.001	0.993 ± 0.001
seq (LFN)	0.949 ± 0.006	0.948 ± 0.012	0.949 ± 0.018	0.901 ± 0.012	0.990 ± 0.002	0.991 ± 0.002
seq-wt (CNN)	0.958 ± 0.007	0.940 ± 0.019	0.978 ± 0.008	0.922 ± 0.012	0.994 ± 0.001	0.994 ± 0.000
seq-wt (LFN)	0.961 ± 0.003	0.963 ± 0.009	0.960 ± 0.012	0.925 ± 0.007	0.994 ± 0.000	0.994 ± 0.000
start (CNN)	0.957 ± 0.002	0.955 ± 0.004	0.959 ± 0.007	0.917 ± 0.005	0.993 ± 0.001	0.993 ± 0.001
start (LFN)	0.956 ± 0.006	0.961 ± 0.006	0.951 ± 0.008	0.914 ± 0.011	0.990 ± 0.002	0.989 ± 0.003

Table C. Classification performance for all eight of our models and three prior methods, presented as mean ± std dev across five replicates.

(LFN) in panel A and bioseq2class (LFN) in panel B. Values close to $\pm\pi$ imply a partial cancellation effect when filtered representations are added to the residual term at the end of each LFNet layer. Bioseq2seq has learned to suppress periodic signals besides 3-nt periodicity while modulating the critical 3-nt signal to a lesser extent. The bioseq2class (LFN) filters show less discrimination between 3-nt and the remainder in terms of phase activity.

C Interpreting encoder-decoder attention

We built nucleotide-resolution metagenes for encoder-decoder attention (EDA) by aligning the attention distributions of each transcript relative to its start codon or AUG of the longest ORF. We used Welch's

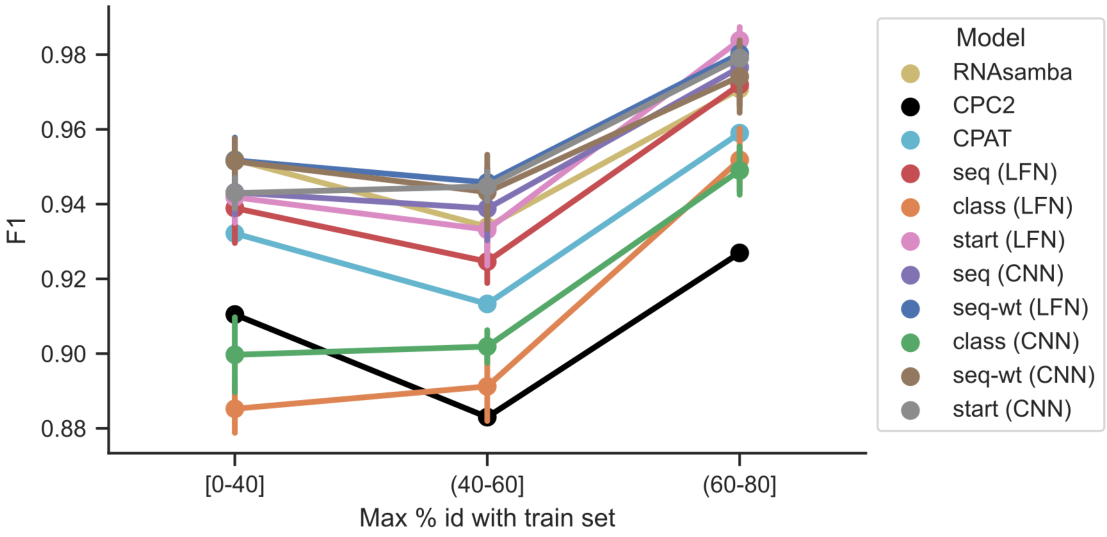

Figure B. F1 results stratified according to maximum similarity to the training set. Results are grouped into similarity bins according to the maximum Needleman-Wunsch alignment identity found for a test set RNA against the training set.

method for spectral density estimation to decompose the attention metagenes into a density over a range of frequencies, plotting the resuling power spectrum decompositions (PSD) in Fig E. Respectively, panels A-D contain PSDs for: bioseq2seq-wt (LFN) on mRNAs, bioseq2class (LFN) on mRNAs, bioseq2seq-wt (LFN) on lncRNAs, and bioseq2class (LFN) on lncRNAs. Bioseq2seq-wt distinguishes the two classes more clearly, with the lncRNA power spectrum containing wavelet-like shapes centered around the $\frac{1}{3}$ Hz and closer to zero. The bioseq2class attention values for lncRNAs simply lead to a somewhat noisier $\frac{1}{3}$ Hz peak than in in mRNAs.

We also binned and averaged encoder-decoder attention (EDA) values within functional regions in the same fashion as previously described for ISM and MDIG. The resulting metagenes are conceptually distinct from those calculated at nucleotide-resolution because the averaging process clarifies positional trends by drowning out the 3-nt periodicity that dominates at nucleotide-resolution. Plots of bin-based metagenes for bioseq2seq-wt (LFN) are shown for every attention head, with heads from the lower decoder layer in panel E and those from the higher decoder in panel F. With the caveat that LFNet encoder embeddings integrate contextual information from many different input positions, we observed that one head from Layer 0 (head 1) placed the bulk of its attention close to the position of the start codon mRNAs but devoted much less attention to the equivalent start region of the longest ORF for lncRNAs. In head 7 from the same layer, this emphasis is flipped, with the lncRNA start position having

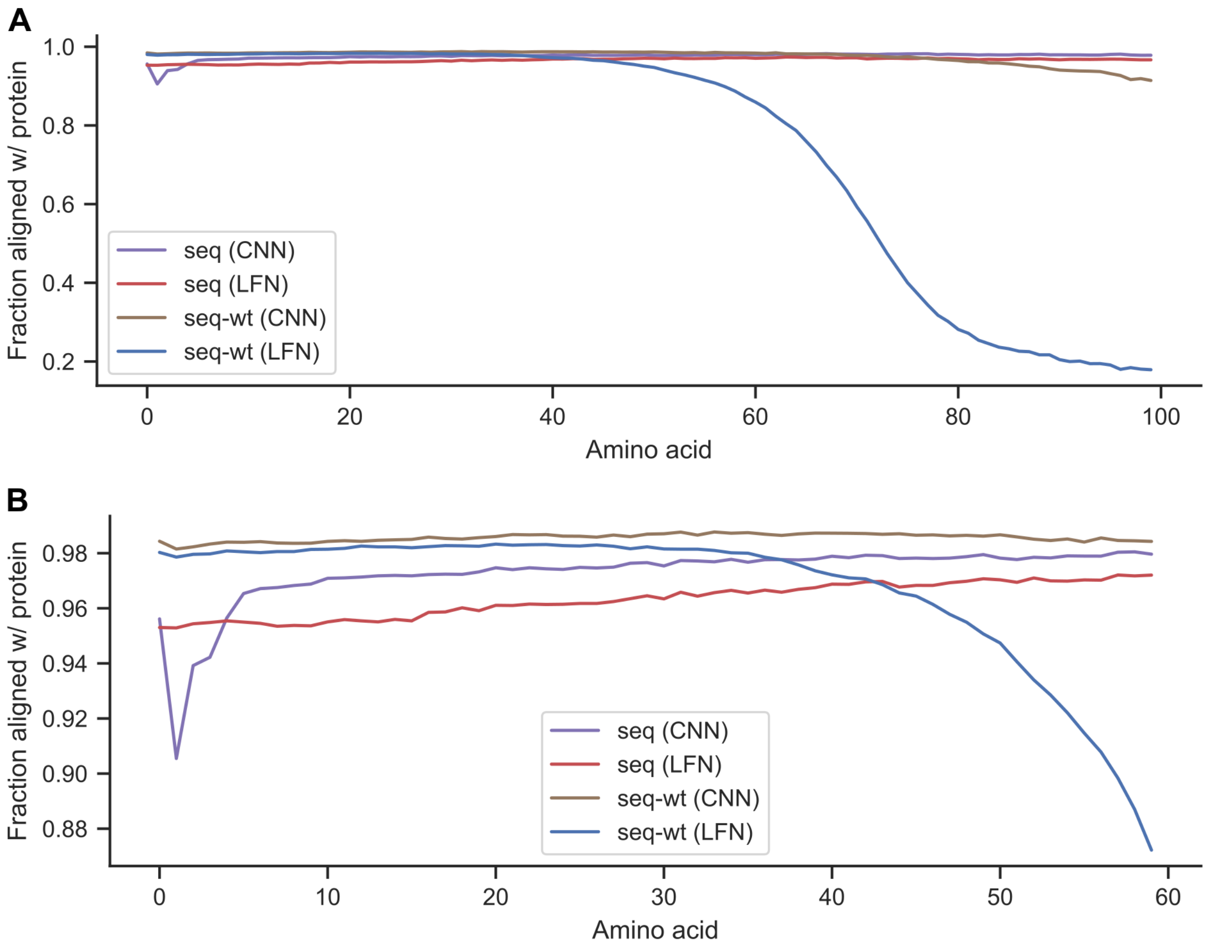

Figure C. Fraction of predicted amino acids aligned to the ground truth by position. (A) All bioseq2seq variants up to 100 codons. (B) The same data zoomed in to 60 amino acids.

a much stronger attention peak. While most layers place attention on start regions and to a lesser extent stop regions, other distributions are shaped more like a bell somewhere along the CDS and a few mostly reflect positional trends. The distinctions between mRNAs and lncRNAs on the metagene plots appear somewhat reduced in Layer 1, which could speak to how the model integrates information hierarchically.

D Data subsampling

To explore feature consistency across model types and replicates while keeping computational costs manageable, we restricted several experiments to a subset of our testing data consisting of the transcripts with verified status in RefSeq. For the experiment described in section 2.6 of the main text, we

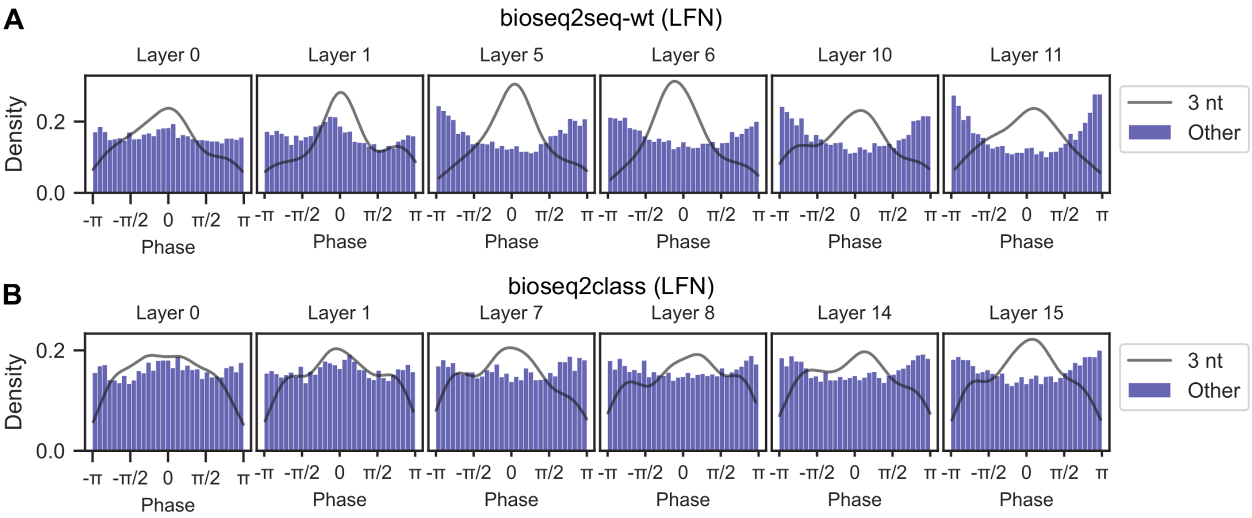

Figure D. LFNet filter phase histograms. (A) Density of phase values for selected layers of bioseq2seq-wt (LFN), with 3-nt as a kernel density plot denoted by the black line and the remaining frequencies as a purple histogram. (B) The same as panel A for bioseq2class (LFN).

took all 220 transcripts with 'NR' ids in the test set and sampled an equal amount of transcripts with 'NM' ids. This is referred to here and in the main text as the "verified test set". The verified validation set used to tune the β value of MDIG in section 2.6 was created using the same criteria.

In several experiments we analyzed transcripts by functional region – CDS and UTRs for mRNAs or the longest ORF and regions upstream and downstream for lncRNAs. For the bin-based metagenes, we used 25 positional bins for each functional region, which led us to require that every functional region be at least 25 nt in length. For the full test set, 998 mRNAs and 1645 lncRNAs met these criteria. For the verified test set these numbers were 93 mRNAs and 125 lncRNAs.

E Gradient-based mutation effect prediction

For all mutagenesis experiments, we used the function $F = l_{\langle PC \rangle} - l_{\langle NC \rangle}$, where l_c is the neural network activation before the final softmax layer for class c. These activations are also called the logits and express finer differences than the post-softmax probabilities. Our choice of F ensures that evidence from both RNA classes contributes to gradient-based attributions.

All our gradient-based attributions are calculated at the one-hot level, but PyTorch sequence models, including ours, are commonly trained with an embedding layer based on a dictionary-like object. To introduce the one-hot representation into the computational graph at inference time, the weights matrix

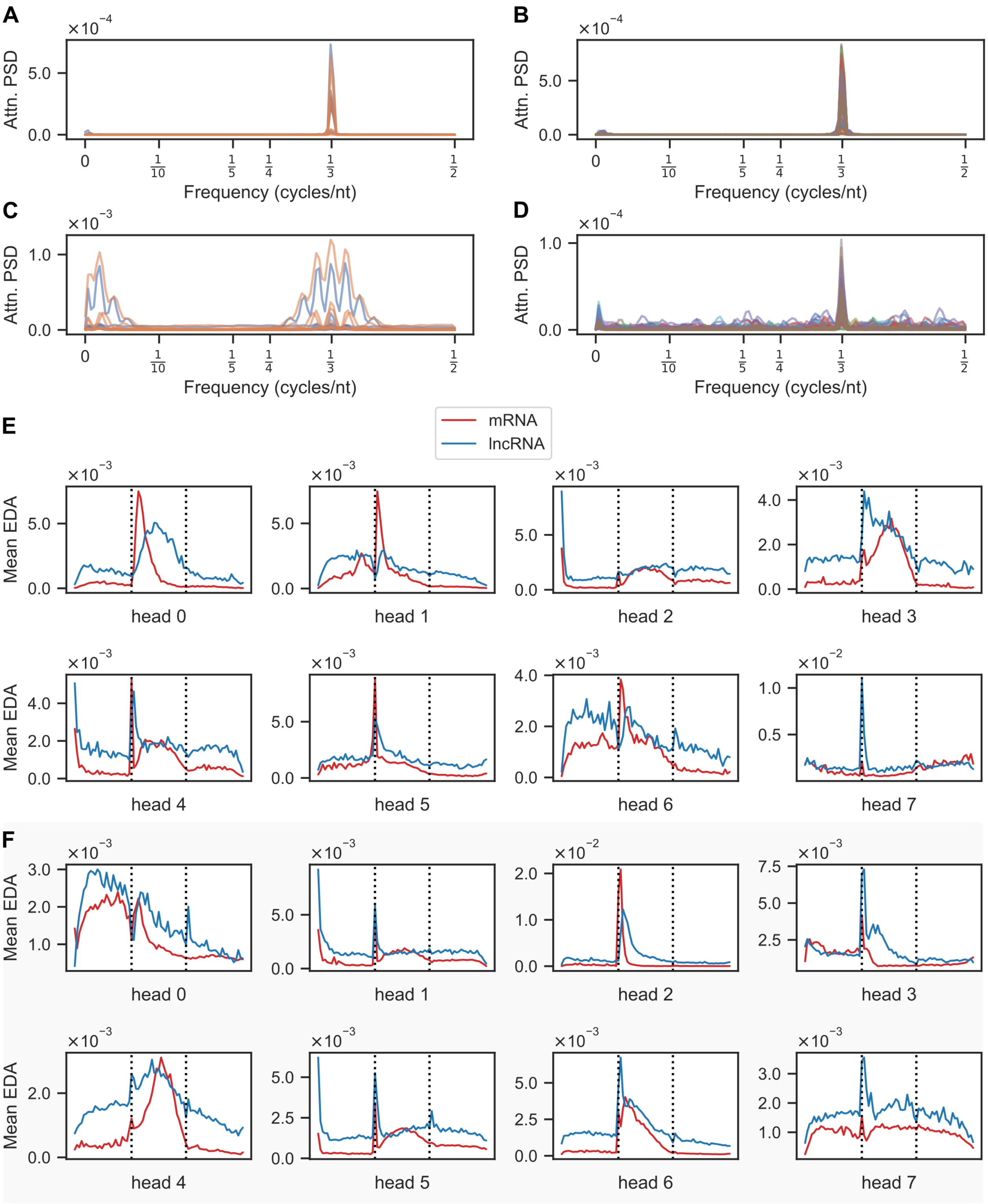

Figure E. Analysis of encoder-decoder attention (EDA). (A) Power spectrum decomposition of EDA metagenes for mRNAs on bioseq2seq-wt (LFN). Equivalent nucleotide positions relative to the start codon in mRNAs were aligned and corresponding attention scores from each attention head were averaged to form the nucleotide-resolution metagene. (B) The same as panel A using encoder-decoder attention from bioseq2class (LFN) (C). Power spectrum decomposition for lncRNAs aligned relative to the AUG of the longest ORF on bioseq2seq-wt (LFN). (D) The same as panel C using encoder-decoder attention from bioseq2class (LFN). (E) EDA from Layer 0 of bioseq2seq-wt (LFN), the lower in the decoder stack, with attention values averaged within 25 positional bins for each functional region. First and last bins of the CDS or longest ORF marked with dotted vertical lines. (F) EDA metagenes from Layer 1, the higher decoder in bioseq2seq-wt (LFN).

object from the torch.nn.Embedding layer is extracted and the input tensors are converted to a one-hot encoding. An explicit matrix multiplication between the one-hot encoding and the embedding weights preserves the behavior of the Embedding layer while enabling differentiation with respect to the one-hot vectors.

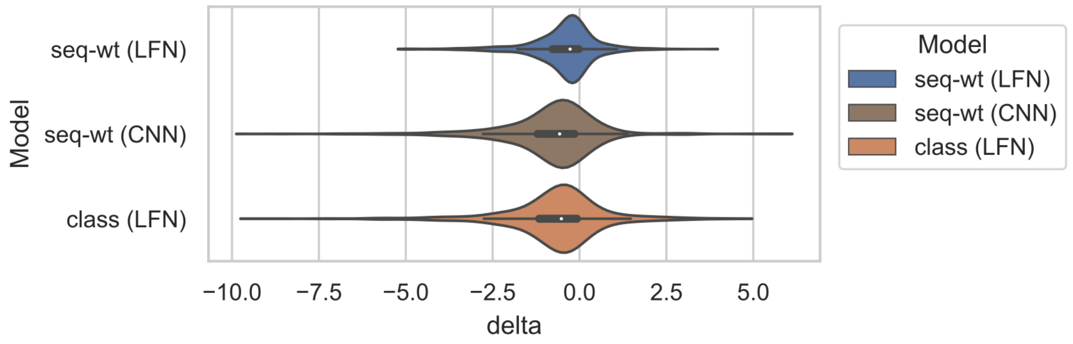

Figure F. Distribution of ΔS scores from replacing mRNA 5' UTRs lacking an upstream ORF with 5' UTRs containing a uORF.

E.1 Mutation Directed Integrated Gradients

MDIG is intended to balance the competing goals of (1) accumulating global information correlated with point mutations and (2) maintaining distance from the uninformative baseline poly(b), while the linear IG interpolation permits parallelism in the sequence dimension. Integrated Gradients satisfies the property $\sum_i \sum_j IG_{ij}(x, x') = F(x) - F(x')$ [6]. Since we are seeking to relate IG scores to $3L$ single-nucleotide variants rather than the baseline x' itself, this identity is of reduced importance compared to typical IG use cases. Still, we used this property to compute approximation error and inform our selection of 32 numerical integration steps for the experiments in the main text, which also provided for adequate convergence of IG values.

A possible explanation for the values of β that proved best during tuning MDIG is that at $\beta < \frac{1}{2}$, the interpolated embedding $Embed(\beta \cdot \text{poly(b)} + (1 - \beta) \cdot x)$ is row-wise closer to $Embed(x)$ rather than $Embed(\text{poly(b)})$ by Euclidean distance. In other words, the closest embedding of any discrete/non-interpolated sequence prior to this point is the original sequence, so the accumulated gradients maintain a connection to the original sequence, whereas at $\beta > \frac{1}{2}$, the embedding is closer to the poly(b) baseline. Proximity of embeddings by Euclidean distance does not ensure any particular relationship between their network outputs, but we found empirically that MDIG can correlate quite well with ISM for

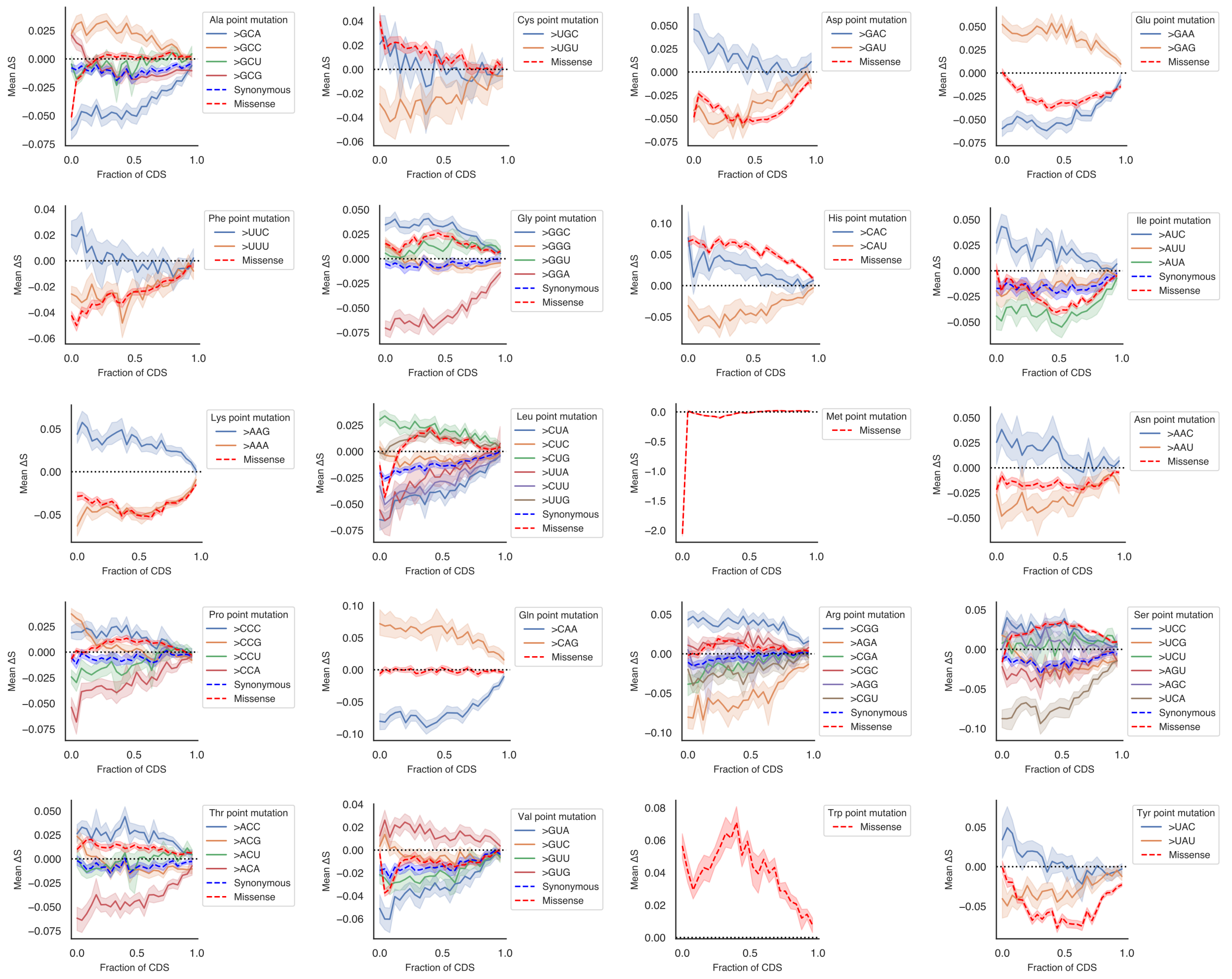

Figure G. Plots of ISM metagenes for all twenty amino acids. Mean ΔS is shown for 25 positional bins across mRNA CDS regions. Mutations are listed based on the resulting codon. The red line represents the average across all missense/non-synonymous mutations. For amino acids with more than two codons, the blue dashed line depicts the average synonymous mutation for comparison.

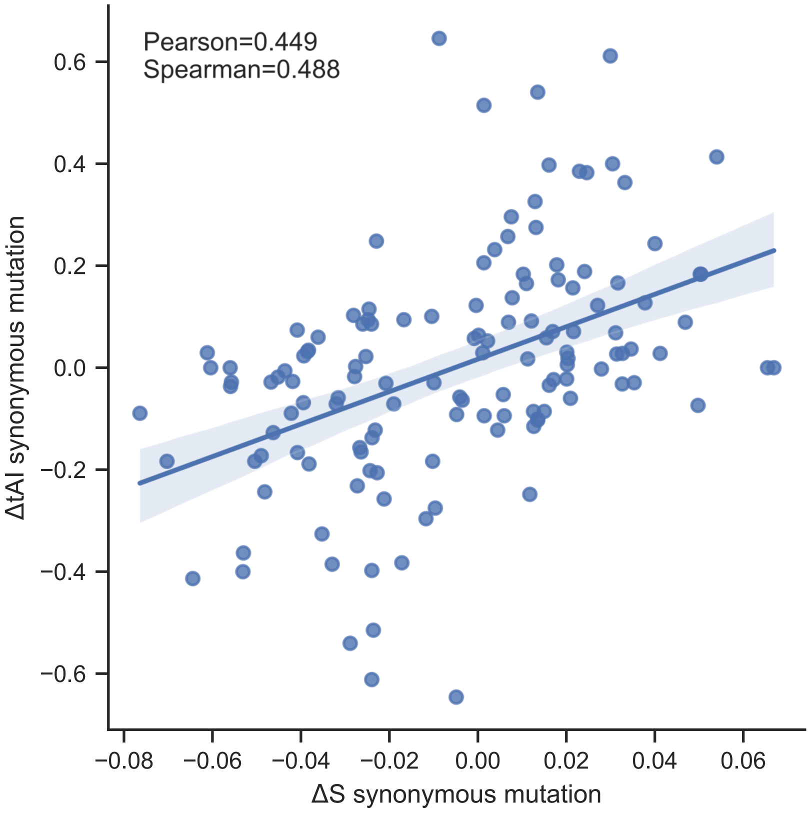

Figure H. Correlation between average ΔS for synonymous codon pairs and differences in tRNA Adaptation Index (tAI) for the same codon pairs, using data from [5]

certain models like bioseq2class and pick up the most important features for the bioseq2seq-wt (LFN) model.

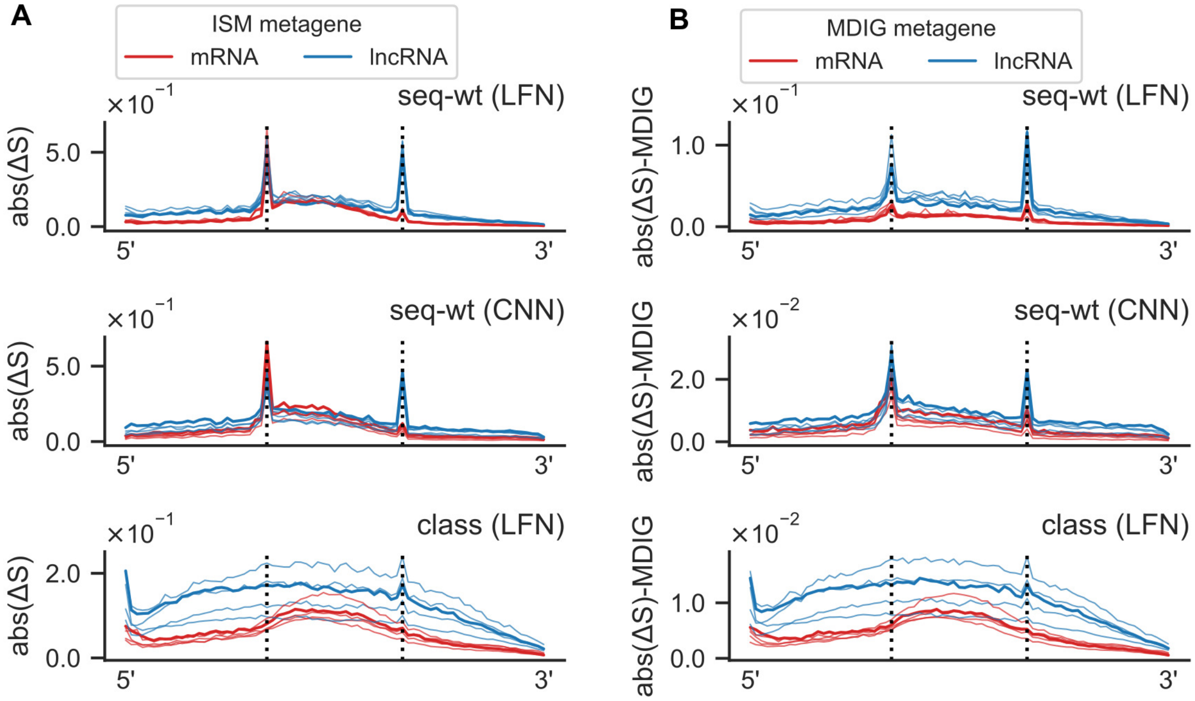

Figure I. Extended results from best settings for MDIG. (A) Plot of bin-based metagene for bioseq2seq-wt (LFN) and (CNN) and bioseq2class (LFN) using ISM, reproduced from the main text. (B) Plot of bin-based metagene for bioseq2seq-wt (LFN) using MDIG-0.25 and bioseq2seq-wt (CNN) and bioseq2class (LFN) both using MDIG-0.1.

F Motif comparison

We used TOMTOM [7] with default parameters except with '-norc' added to compare motifs found in control strategy 2 matching ones from the purely random control strategy. Matches at this significance threshold were omitted from our analysis to emphasize those uniquely identifiable with our model-derived importance scores. MDIG motifs were run against RBPs from [8] with three top possible matches shown in Fig J.

G Motivation from Transformer encoder

Earlier in model development, we experimented with transformer neural networks [4] for both the encoder and decoder stacks based on the widespread success of the transformer in natural language

Summary ?

**Name**	RNCMPT00282 (Rbm42)
**Database**	Ray2013_rbp_All_Species.dna_encoded
***p*-value**	4.97e-05
***E*-value**	1.21e-02
***q*-value**	1.19e-02
**Overlap**	8
**Offset**	0
**Orientation**	Normal

Show logo download options

Optimal Alignment ?

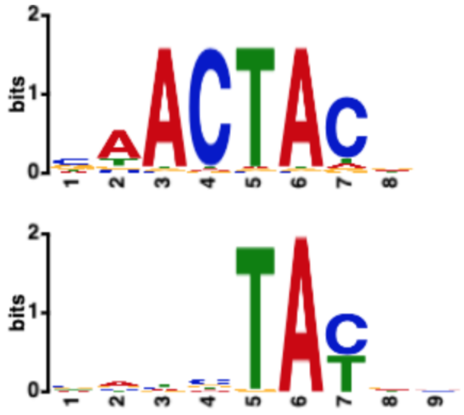

Summary ?

**Name**	RNCMPT00218 (Tb_0218)
**Database**	Ray2013_rbp_All_Species.dna_encoded
***p*-value**	4.38e-04
***E*-value**	1.07e-01
***q*-value**	1.07e-01
**Overlap**	6
**Offset**	1
**Orientation**	Normal

Show logo download options

Optimal Alignment ?

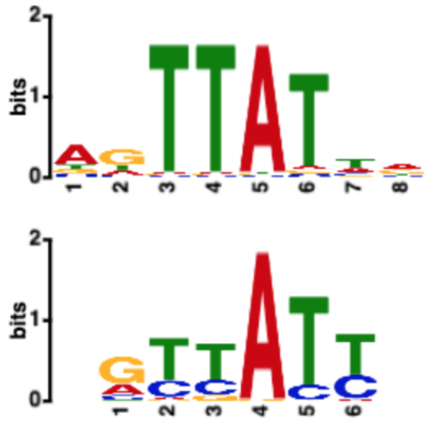

Summary ?

**Name**	RNCMPT00090 (SRSF10)
**Database**	Ray2013_rbp_All_Species.dna_encoded
***p*-value**	2.01e-03
***E*-value**	4.90e-01
***q*-value**	9.61e-02
**Overlap**	6
**Offset**	0
**Orientation**	Normal

Show logo download options

Optimal Alignment ?

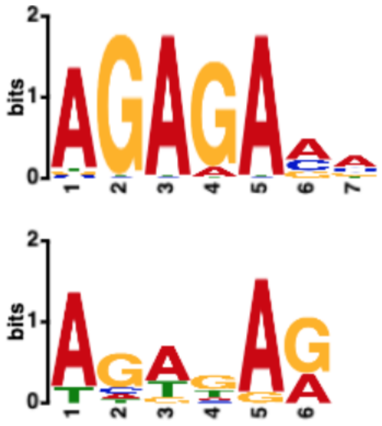

Figure J. Matches with CIS-BP-RNA RNA-binding motifs from [8]

processing. While this architecture successfully learned the genetic code, its classification accuracy lagged noticeably behind the state of the art methods such as RNAsamba. Whereas LocalFilterNet uses a short-time Fourier transform and frequency domain filters, transformers use self-attention to calculate an all-by-all similarity between input embeddings. This $O(N^2)$ complexity of time and especially memory contributed to our inability to train a competitive model with transformers. However, we observed interesting properties of the learned transformer representations that inspired our eventual design of LFNet, particularly a striking dependence on relative positional offsets in self-attention.

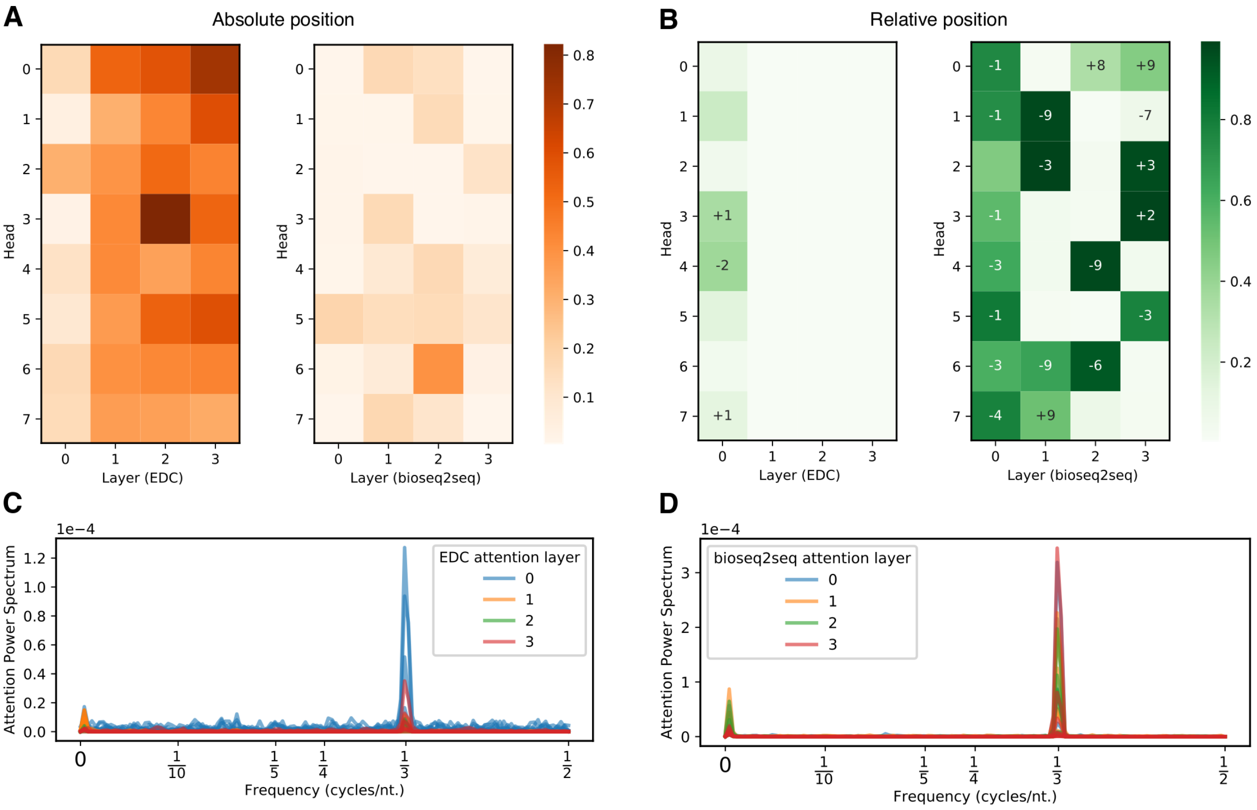

Figure K. Evidence of periodicity from model version using a transformer encoder. (A) Multi-headed self-attention by transformer encoder layer, as a heatmap colored according to the average percentage of input positions in a transcript that maximally attend to a single absolute position. (B) The same as panel A but according to a relative position, with the dominant positional offset listed for each head. (C) Power Spectrum Decomposition for bioseq2seq-Transformer on mRNAs. (D) PSD for bioseq2class-Transformer on mRNAs (formerly known as EDC for Encoder-Decoder Classifier).

For a transcript of length L, a self-attention head produces an $L \times L$ dimensional matrix, with each row containing a probability distribution weighting the local influence of each nucleotide position on each other. For each nucleotide i, we collected its self-attention argmax, recording both its absolute position j and relative positional offset $j - i$. We then determined the mode of these values for the transcript, $mode_{abs}$ or $mode_{rel}$. Finally, we recorded the support for the mode – the percentage of

indexes which equal the modal index n_{abs} or n_{rel}. To summarize the behavior of each self-attention head, we aggregated these transcript-level statistics over every RNA in the test set. Fig K depicts these results, with the cells in the heatmap colored with the average value of n_{abs} or n_{rel}. Whenever the variance of $mode_{rel}$ over all RNAs was less than 1, we included $mode_{rel}$ as an annotation. A dark green cell with label $+1$ therefore denotes a head whose primary behavior is nearly always to pass information from the nucleotide immediately downstream of the current nucleotide.

The moderately high average values of n_{abs} for the attention heads of bioseq2class indicate that a single position within each transcript was often the largest contributor towards updating its encoder representations. By comparison, bioseq2seq devoted little attention to specific loci. However, relative positional heads were much more prevalent in bioseq2seq compared to bioseq2class. Notably, 7/8 heads in Layer 0 of bioseq2seq had a mean value of $n_{abs} > 0.6$ and a zero-variance modal relative offset. Layer3 showed a different pattern, with four heads having mean value of $n_{abs} > 0.9$ and four heads with positional offsets at short distances divisible by three, corresponding to a range of two or three codons away. The prevalence of periodic activity in the learned transformer led us to revisit the importance of the 3-nt periodicity used in many classical gene-finding techniques, and ultimately to modify GlobalFilterNet as the basis for the LocalFilterNet [9].

References

[1] YY Yang et al. TorchAudio: Building Blocks for Audio and Speech Processing. arXiv:2110.15018 [cs, eess]. Feb. 2022.

[2] R Liaw, E Liang, R Nishihara, P Moritz, JE Gonzalez, and I Stoica. Tune: A Research Platform for Distributed Model Selection and Training. arXiv:1807.05118 [cs, stat]. July 2018.

[3] S Falkner, A Klein, and F Hutter. BOHB: Robust and Efficient Hyperparameter Optimization at Scale. en. In: *Proceedings of the 35th International Conference on Machine Learning*. ISSN: 2640-3498. PMLR, July 2018, pp. 1437–1446.

[4] A Vaswani, N Shazeer, N Parmar, J Uszkoreit, L Jones, AN Gomez, Kaiser, and I Polosukhin. Attention is All you Need. In: *Advances in Neural Information Processing Systems*. Vol. 30. Curran Associates, Inc., 2017.

[5] T Tuller, A Carmi, K Vestsigian, S Navon, Y Dorfan, J Zaborske, T Pan, O Dahan, I Furman, and Y Pilpel. An Evolutionarily Conserved Mechanism for Controlling the Efficiency of Protein Translation. *Cell*. **141**: 344–354.

[6] M Sundararajan, A Taly, and Q Yan. Axiomatic Attribution for Deep Networks. *arXiv:1703.01365 [cs]*.

[7] S Gupta, JA Stamatoyannopoulos, TL Bailey, and WS Noble. Quantifying similarity between motifs. *Genome Biology*. **8**: R24.

[8] D Ray et al. A compendium of RNA-binding motifs for decoding gene regulation. *Nature*. **499**: 172–177.

[9] Y Rao, W Zhao, Z Zhu, J Lu, and J Zhou. Global Filter Networks for Image Classification. In: *Advances in Neural Information Processing Systems*. Vol. 34. Curran Associates, Inc., 2021, pp. 980–993.
