# Supplementary material for: Improving deep models of protein-coding potential with a Fourier-transform architecture and machine translation task
Source: PLoS Comput Biol. 2023 Oct 12;19(10):e1011526. doi: 10.1371/journal.pcbi.1011526 (PMC10597526; doi:10.1371/journal.pcbi.1011526)
Supplement: S2 Table — (PDF) [file pcbi.1011526.s003.pdf]

Motif #	Region	Positive Set (sites)	Negative Set (sites)	Pos. Sites	Neg. Sites	Cluster	Logo	Start site in region	Start site in window	Offset from ORF	E-value	p-value	Information
0	ORF	lncRNAs (↑ NC)	lncRNAs (random)	1477/1821 (81.1%)	897/1821 (49.3%)	0	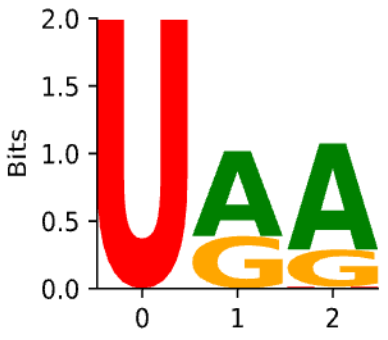	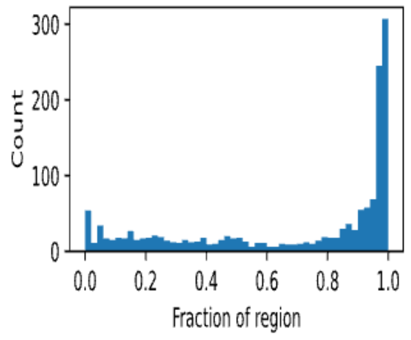	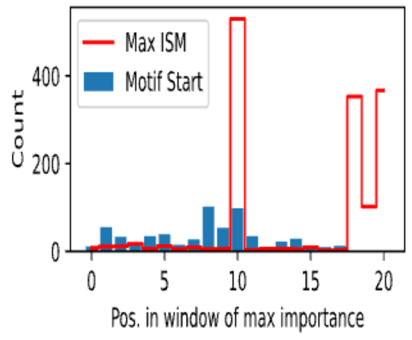	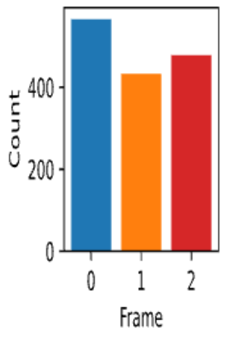	1.72E-10	1.40E-12	4.08
1	ORF	lncRNAs (↑ NC)	mRNAs (↑ NC)	875/1821 (48.1%)	389/1810 (21.5%)	0	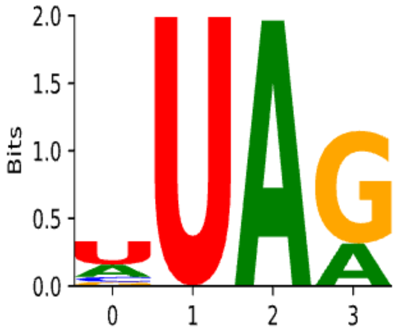	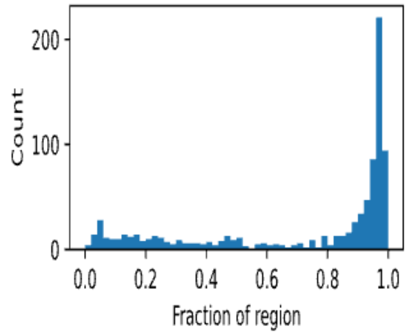	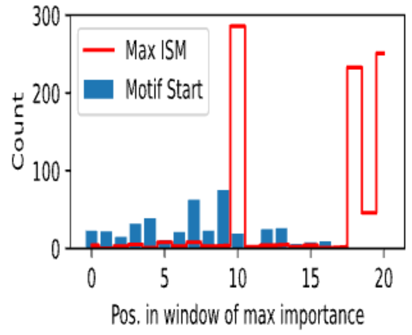	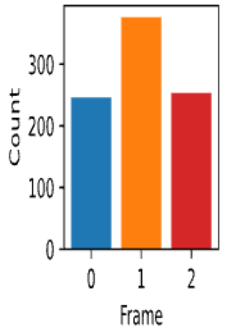	1.60E-07	1.30E-09	5.43
