# Supplementary material for: Improving deep models of protein-coding potential with a Fourier-transform architecture and machine translation task
Source: PLoS Comput Biol. 2023 Oct 12;19(10):e1011526. doi: 10.1371/journal.pcbi.1011526 (PMC10597526; doi:10.1371/journal.pcbi.1011526)
Supplement: S3 Table — (PDF) [file pcbi.1011526.s004.pdf]

Motif #	Region	Positive Set (sites)	Negative Set (sites)	Pos. Sites	Neg. Sites	Cluster	Logo	Start site in region	Start site in window	Offset from ORF	E-value	p-value	Information
0	5-prime	mRNAs (↑ PC)	lncRNAs (↑ PC)	406/1027 (39.5%)	209/1676 (12.5%)	0	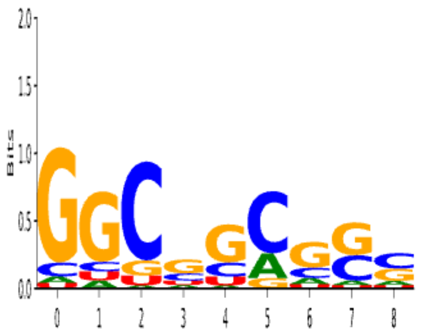	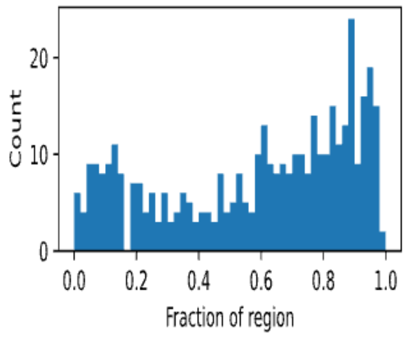	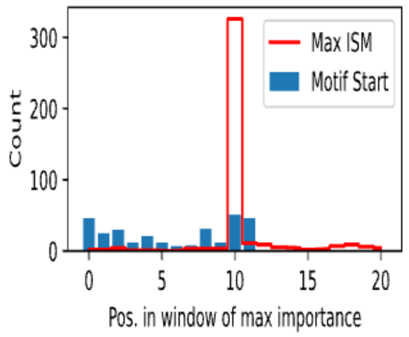	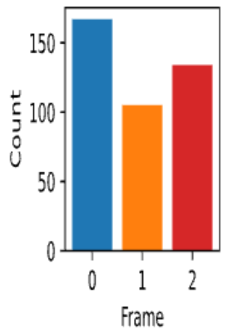	7.32E-04	5.90E-06	5.21
1	ORF	mRNAs (↑ PC)	mRNAs (random)	762/1810 (42.1%)	191/1810 (10.6%)	1	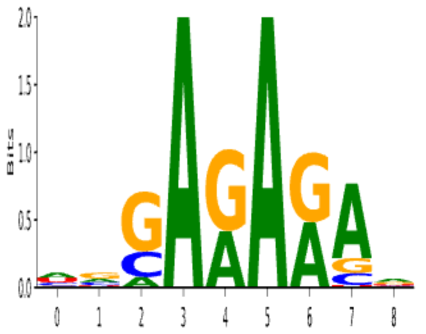	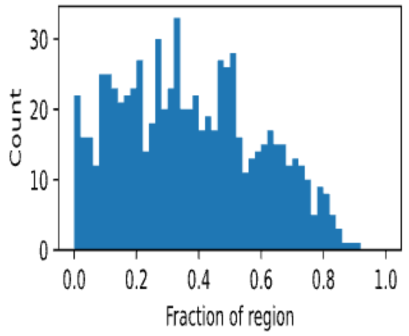	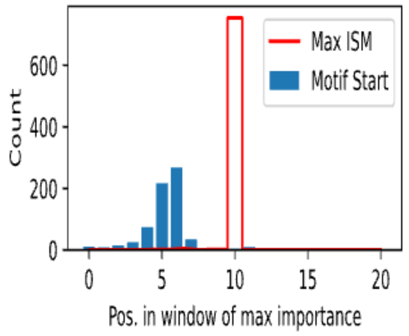	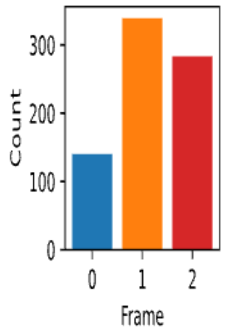	9.80E-12	7.90E-14	7.78
