# Supplementary material for: Improving deep models of protein-coding potential with a Fourier-transform architecture and machine translation task
Source: PLoS Comput Biol. 2023 Oct 12;19(10):e1011526. doi: 10.1371/journal.pcbi.1011526 (PMC10597526; doi:10.1371/journal.pcbi.1011526)
Supplement: S4 Table — (PDF) [file pcbi.1011526.s005.pdf]

Motif #	Region	Positive Set (sites)	Negative Set (sites)	Pos. Sites	Neg. Sites	Cluster	Logo	Start site in region	Start site in window	Offset from ORF	E-value	p-value	Information
0	3-prime	mRNAs (↑ PC)	mRNAs (random)	13594/18151 (74.9%)	9624/18151 (53.0%)	0	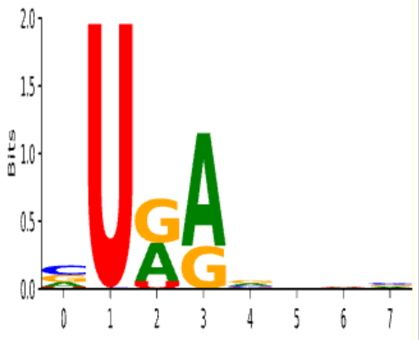	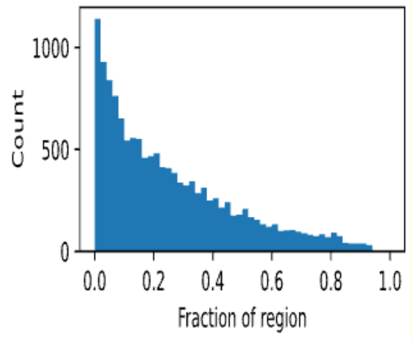	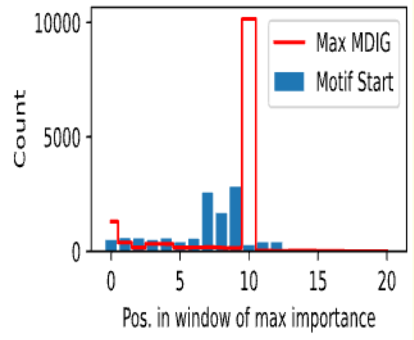	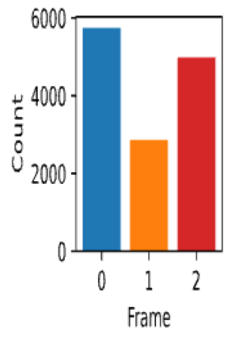	1.24E-41	5.70E-44	4.08
1	3-prime	mRNAs (↑ NC)	mRNAs (random)	10130/18151 (55.8%)	6774/18151 (37.3%)	1	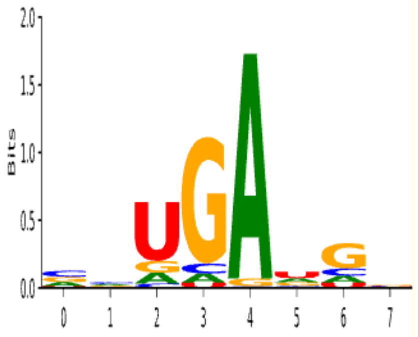	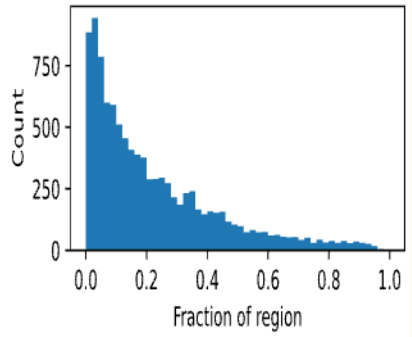	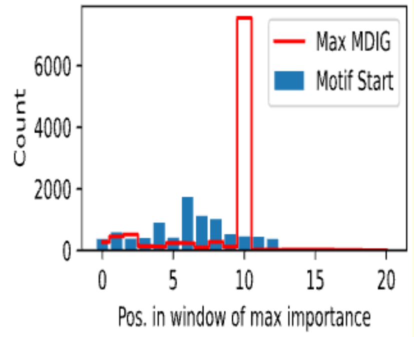	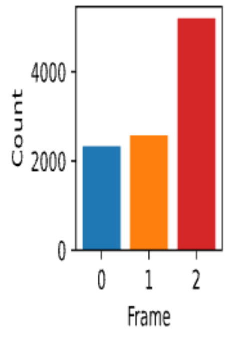	1.93E-33	8.90E-36	4.12
2	3-prime	lncRNAs (↑ PC)	lncRNAs (random)	17218/22525 (76.4%)	12569/22525 (55.8%)	0	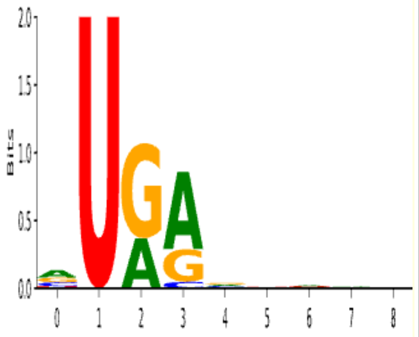	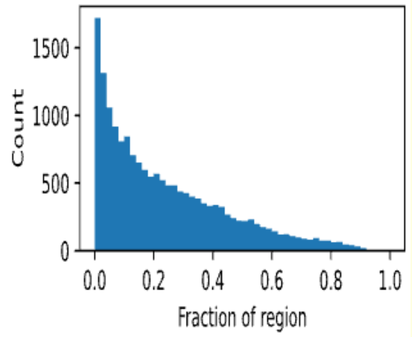	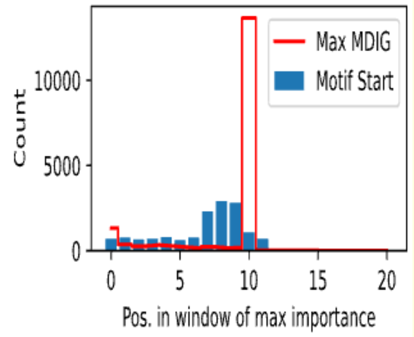	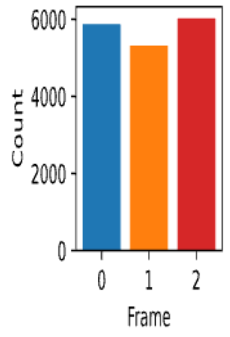	2.02E-46	9.30E-49	4.15
3	3-prime	lncRNAs (↑ NC)	lncRNAs (random)	19226/22525 (85.4%)	12459/22525 (55.3%)	0	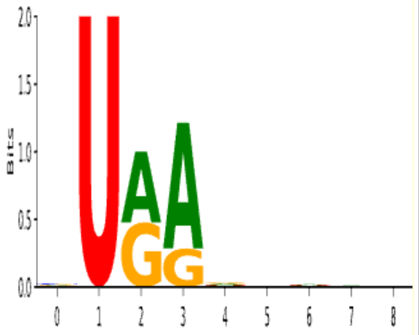	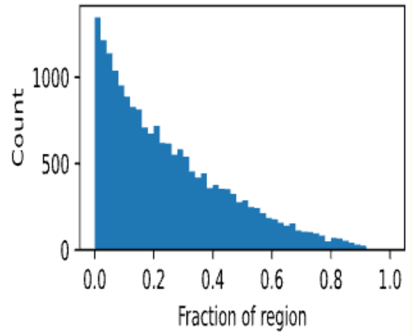	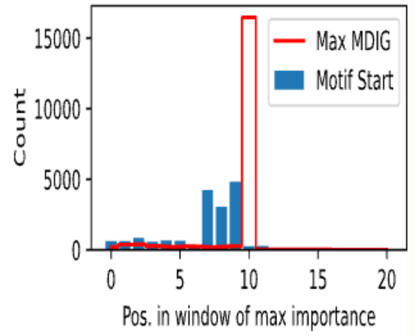	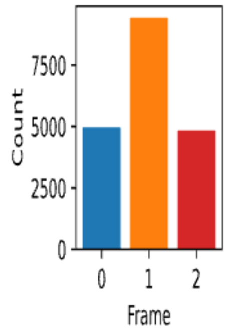	7.60E-95	3.50E-97	4.32

Motif #	Region	Positive Set (sites)	Negative Set (sites)	Pos. Sites	Neg. Sites	Cluster	Logo	Start site in region	Start site in window	Offset from ORF	E-value	p-value	Information
4	5-prime	mRNAs (↑ PC)	mRNAs (random)	9550/15842 (60.3%)	6073/15842 (38.3%)	2	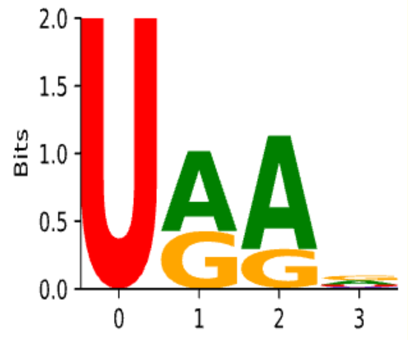	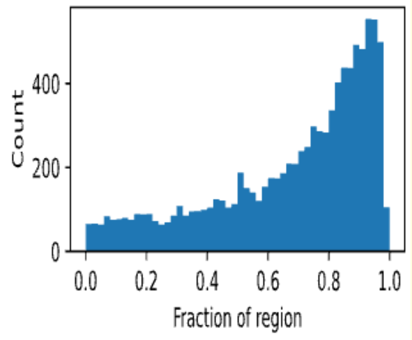	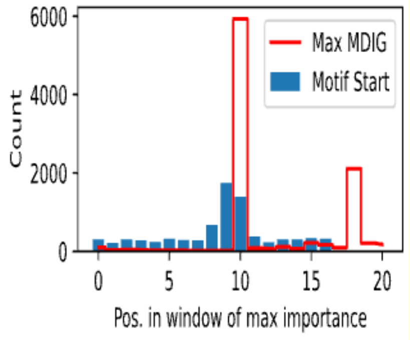	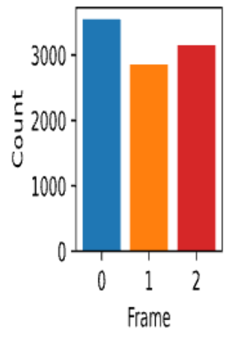	2.17E-22	1.00E-24	4.24
5	5-prime	mRNAs (↑ NC)	mRNAs (random)	4181/15842 (26.4%)	1029/15842 (6.5%)	3	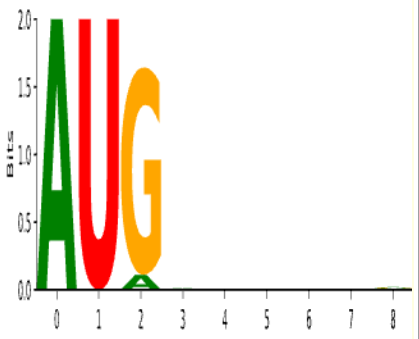	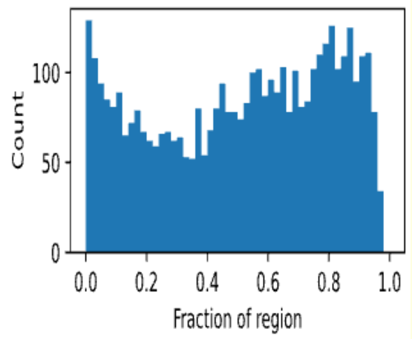	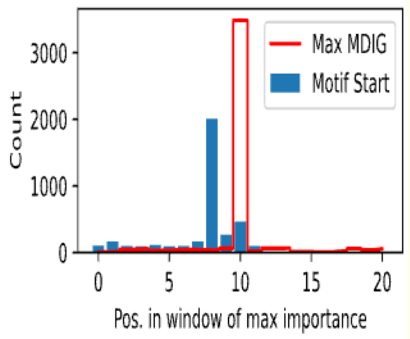	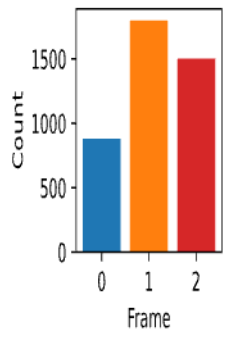	1.06E-41	4.90E-44	5.69
6	5-prime	mRNAs (↑ NC)	mRNAs (random)	6748/15842 (42.6%)	5716/15842 (36.1%)	2	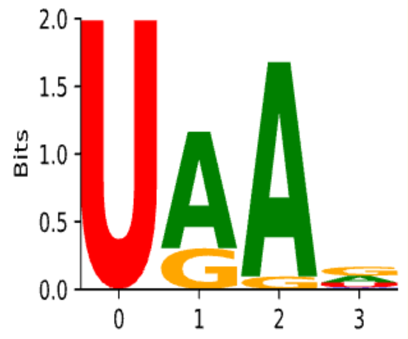	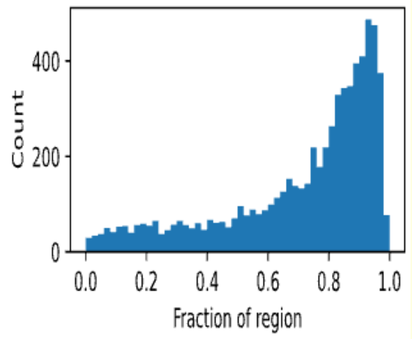	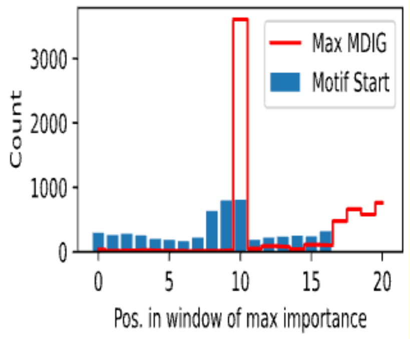	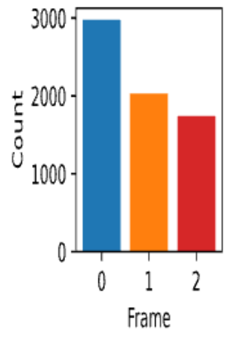	4.34E-05	2.00E-07	5.00
7	5-prime	lncRNAs (↑ PC)	lncRNAs (random)	11844/23017 (51.5%)	3338/23017 (14.5%)	3	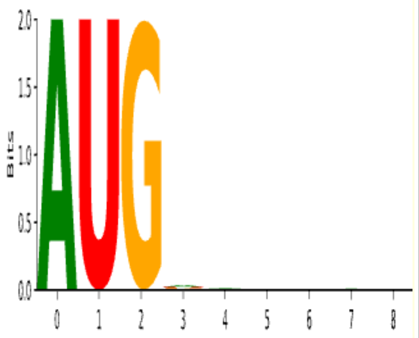	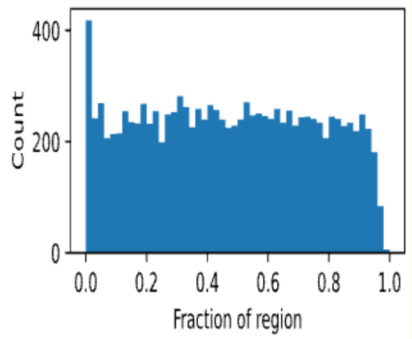	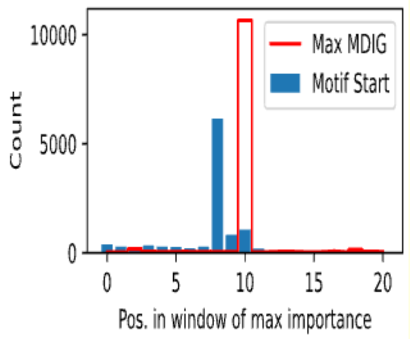	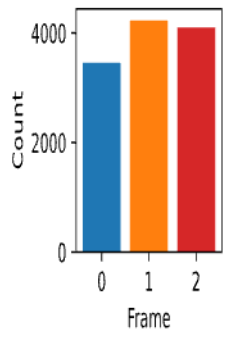	3.47E-175	1.60E-177	6.06

Motif #	Region	Positive Set (sites)	Negative Set (sites)	Pos. Sites	Neg. Sites	Cluster	Logo	Start site in region	Start site in window	Offset from ORF	E-value	p-value	Information
8	5-prime	lncRNAs (↑ NC)	lncRNAs (random)	19183/23017 (83.3%)	10871/23017 (47.2%)	0	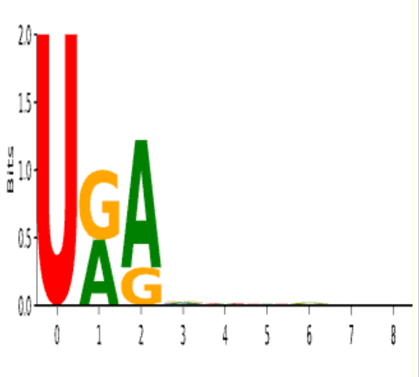	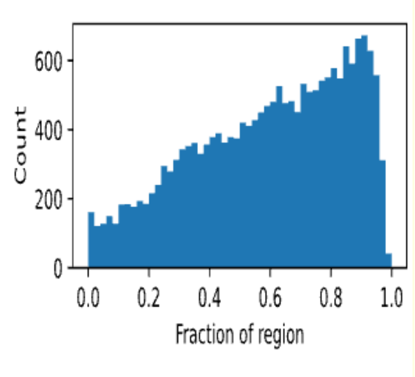	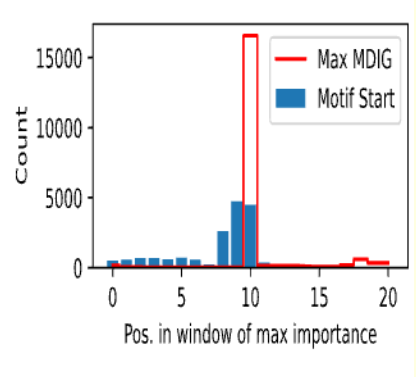	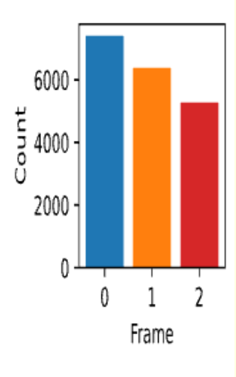	1.74E-153	8.00E-156	4.31
9	5-prime	lncRNAs (↑ PC)	mRNAs (↑ PC)	9849/23017 (42.8%)	4865/15842 (30.7%)	4	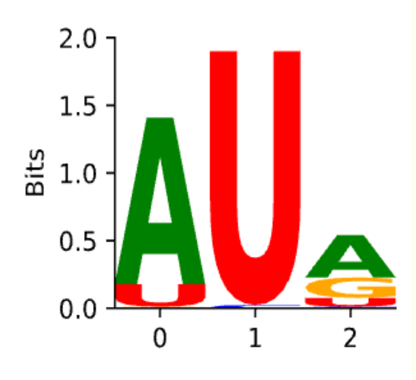	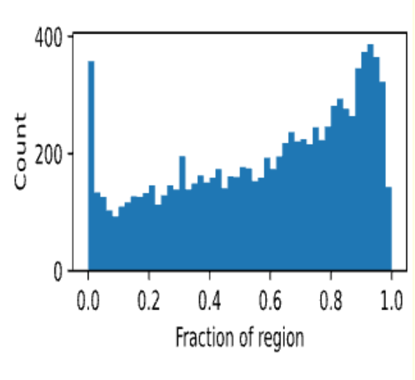	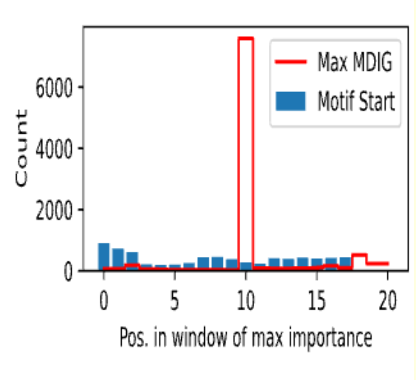	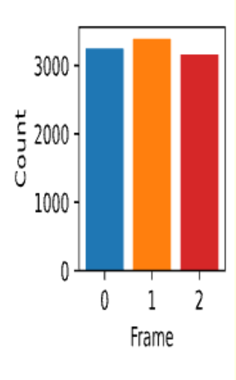	4.56E-14	2.10E-16	3.85
10	ORF	mRNAs (↑ PC)	mRNAs (random)	17190/26039 (66.0%)	5203/26039 (20.0%)	3	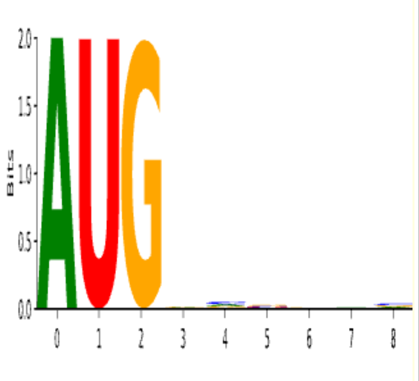	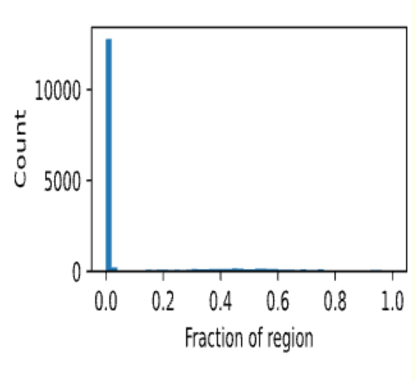	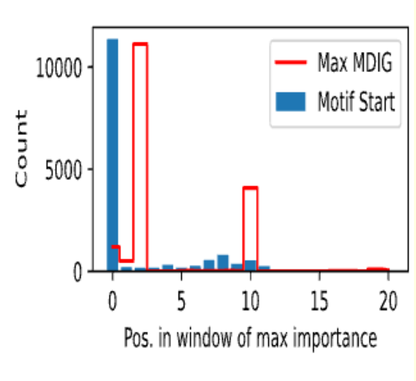	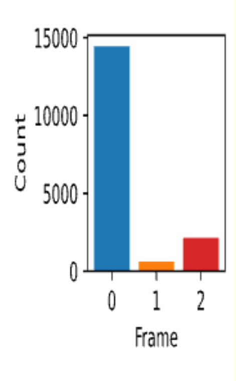	6.73E-236	3.10E-238	6.14
11	ORF	mRNAs (↑ NC)	mRNAs (random)	20600/26039 (79.1%)	13100/26039 (50.3%)	4	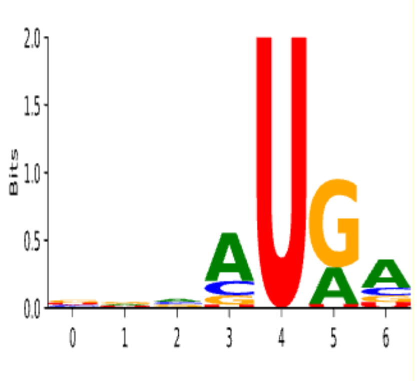	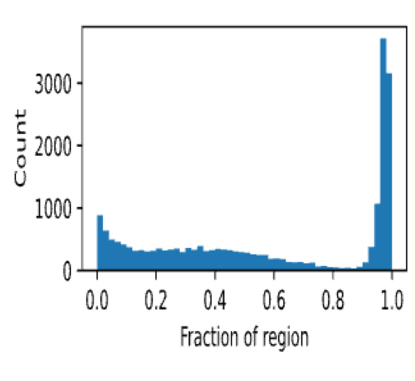	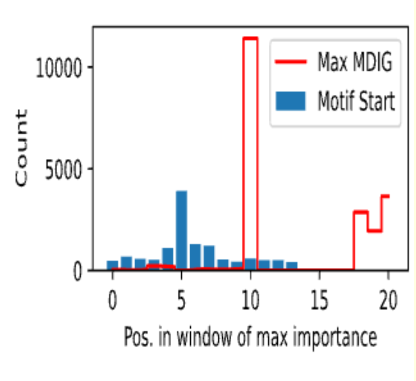	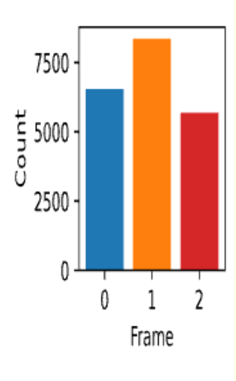	4.99E-104	2.30E-106	4.04

Motif #	Region	Positive Set (sites)	Negative Set (sites)	Pos. Sites	Neg. Sites	Cluster	Logo	Start site in region	Start site in window	Offset from ORF	E-value	p-value	Information
12	ORF	mRNAs (↑ NC)	mRNAs (random)	2190/26039 (8.4%)	1241/26039 (4.8%)	5	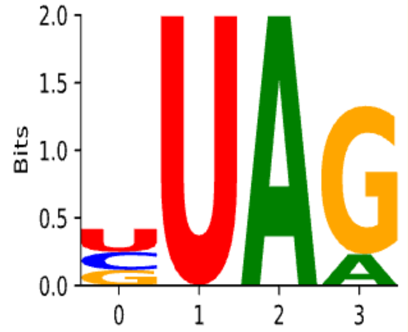	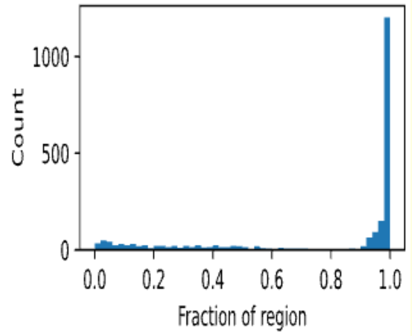	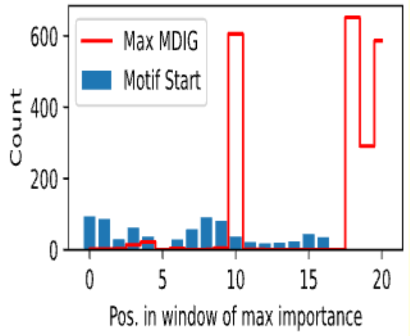	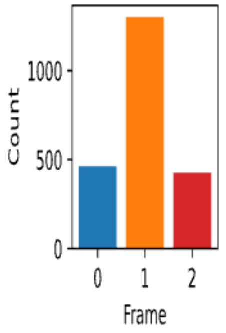	5.21E-07	2.40E-09	5.73
13	ORF	lncRNAs (↑ PC)	lncRNAs (random)	18332/24881 (73.7%)	5037/24881 (20.2%)	3	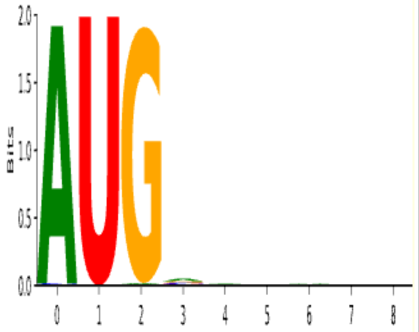	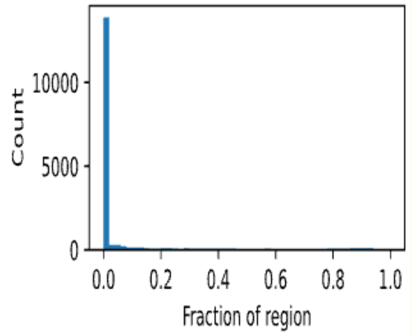	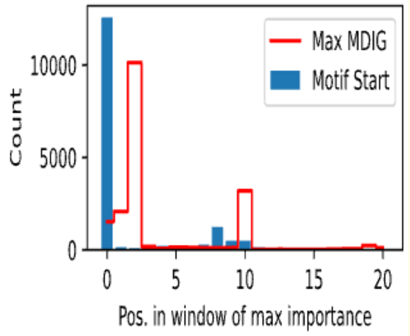	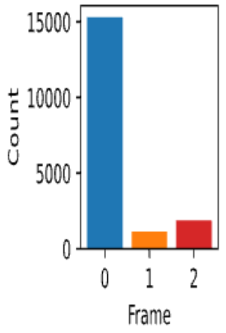	1.07E-320	4.94E-323	5.90
14	ORF	lncRNAs (↑ NC)	lncRNAs (random)	22583/24881 (90.8%)	11548/24881 (46.4%)	2	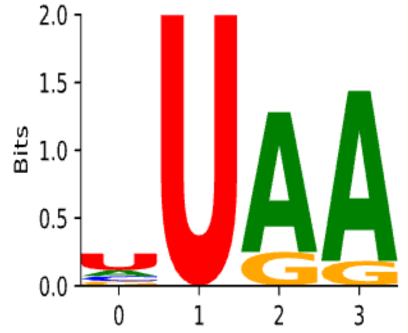	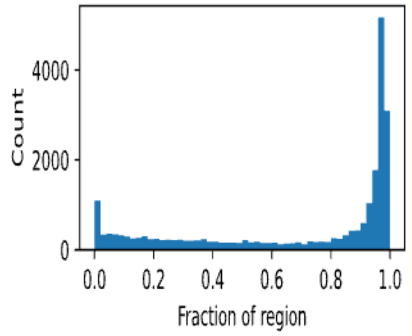	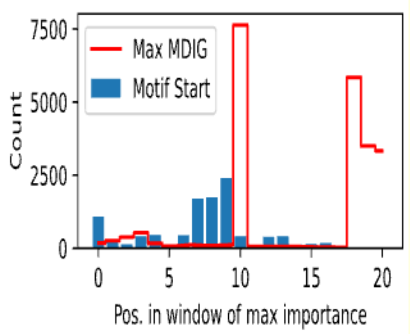	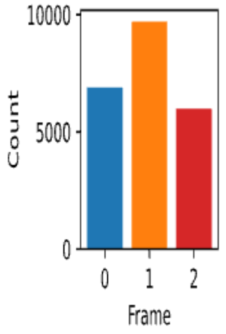	1.61E-268	7.40E-271	4.95
15	ORF	mRNAs (↑ NC)	lncRNAs (↑ NC)	2498/26039 (9.6%)	1423/24881 (5.7%)	4	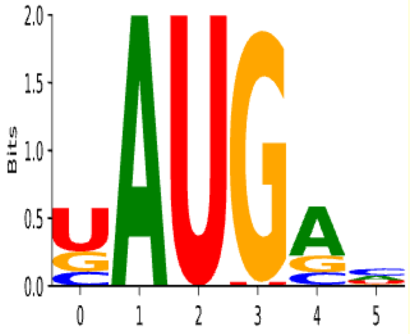	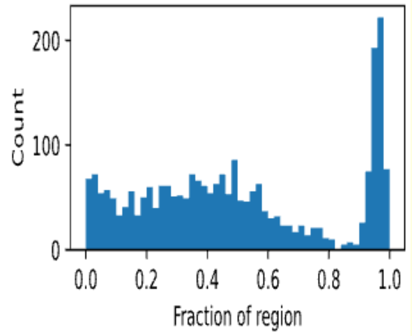	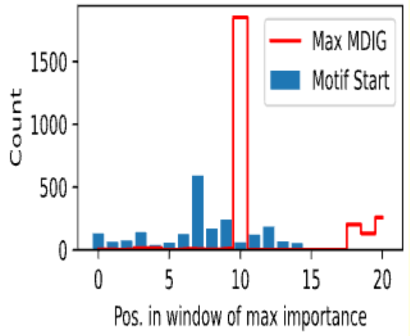	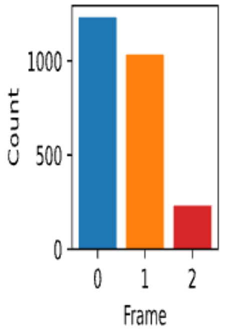	4.56E-07	2.10E-09	7.16
