# Supplementary material for: Improving deep models of protein-coding potential with a Fourier-transform architecture and machine translation task
Source: PLoS Comput Biol. 2023 Oct 12;19(10):e1011526. doi: 10.1371/journal.pcbi.1011526 (PMC10597526; doi:10.1371/journal.pcbi.1011526)
Supplement: S5 Table — (PDF) [file pcbi.1011526.s006.pdf]

Motif #	Region	Positive Set (sites)	Negative Set (sites)	Pos. Sites	Neg. Sites	Cluster	Logo	Start site in region	Start site in window	Offset from ORF	E-value	p-value	Information
0	3-prime	mRNAs (↑ PC)	mRNAs (random)	12697/18151 (70.0%)	10372/18151 (57.1%)	0	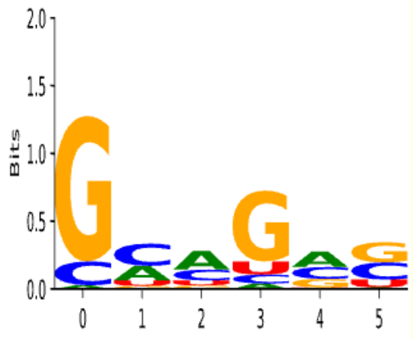	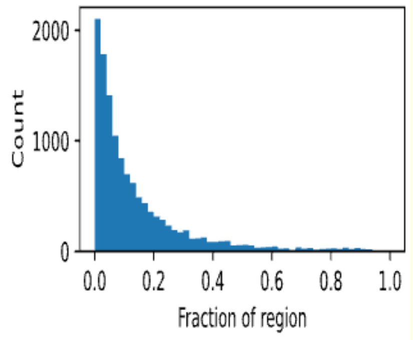	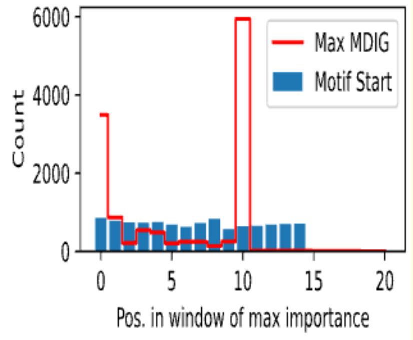	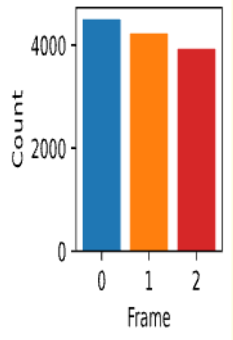	1.79E-14	7.20E-17	3.24
1	3-prime	mRNAs (↑ NC)	mRNAs (random)	9507/18151 (52.4%)	7189/18151 (39.6%)	0	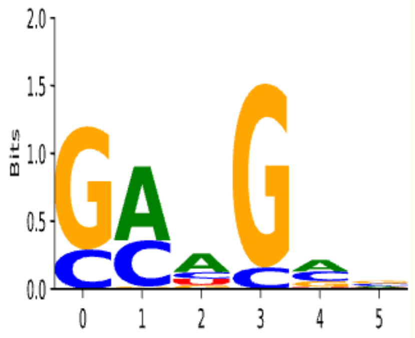	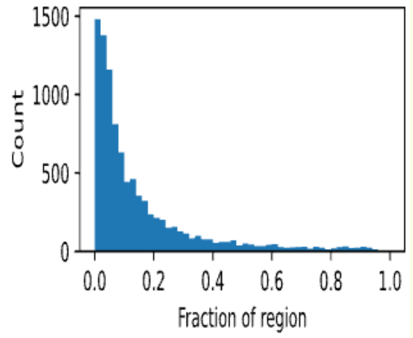	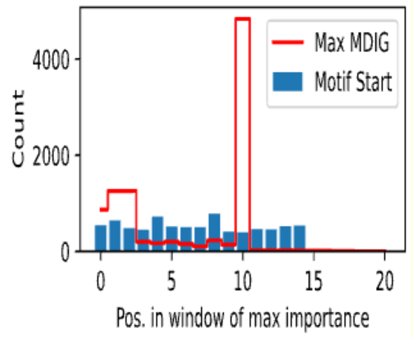	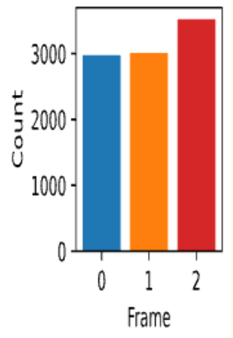	1.72E-09	6.90E-12	4.15
2	5-prime	mRNAs (↑ NC)	mRNAs (random)	5146/15842 (32.5%)	3245/15842 (20.5%)	1	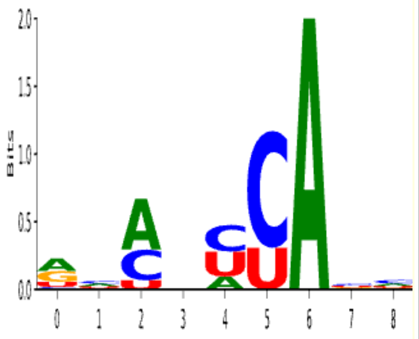	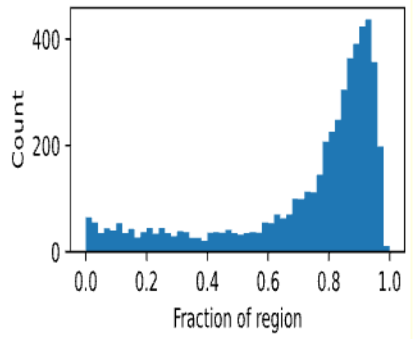	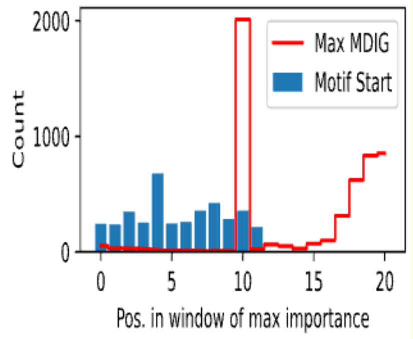	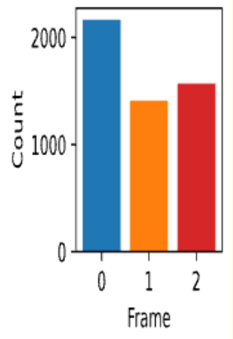	4.23E-08	1.70E-10	4.72
3	5-prime	lncRNAs (↑ PC)	lncRNAs (random)	11475/23017 (49.9%)	8674/23017 (37.7%)	2	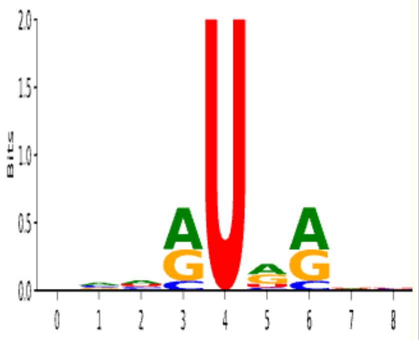	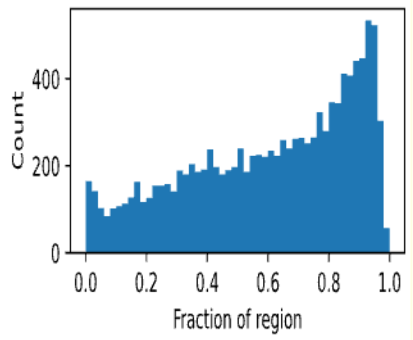	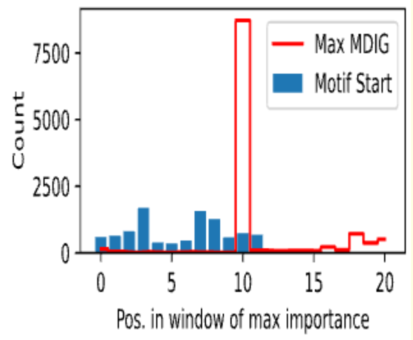	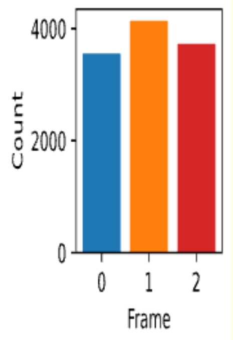	2.09E-14	8.40E-17	3.59

Motif #	Region	Positive Set (sites)	Negative Set (sites)	Pos. Sites	Neg. Sites	Cluster	Logo	Start site in region	Start site in window	Offset from ORF	E-value	p-value	Information
4	5-prime	lncRNAs (↑ NC)	lncRNAs (random)	13009/23017 (56.5%)	10877/23017 (47.3%)	3	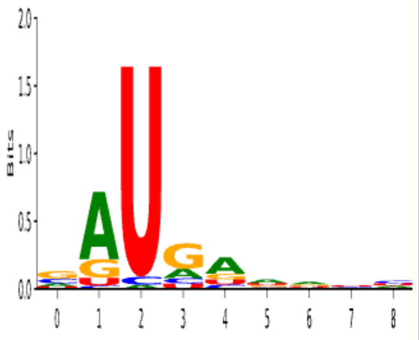	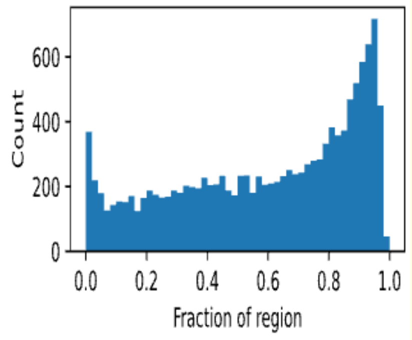	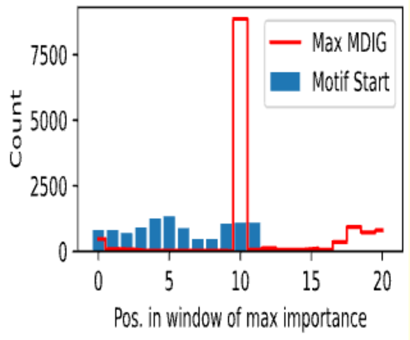	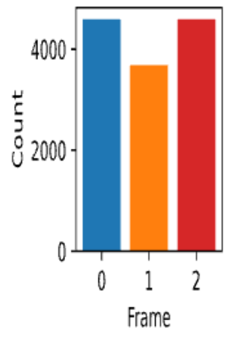	1.82E-05	7.30E-08	3.27
5	ORF	mRNAs (↑ PC)	mRNAs (random)	14004/26039 (53.8%)	4980/26039 (19.1%)	4	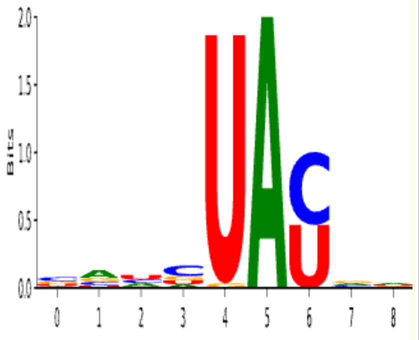	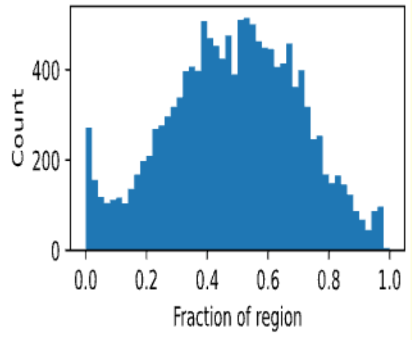	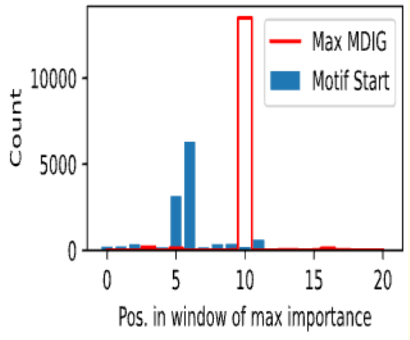	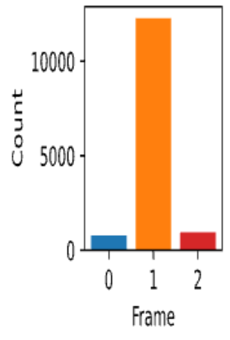	2.32E-137	9.30E-140	5.43
6	ORF	mRNAs (↑ PC)	mRNAs (random)	7961/26039 (30.6%)	6397/26039 (24.6%)	3	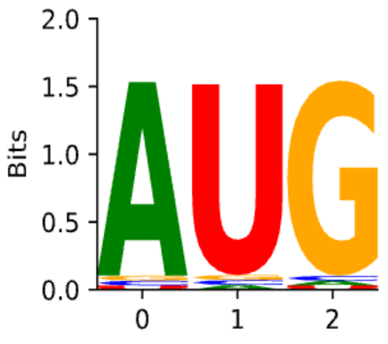	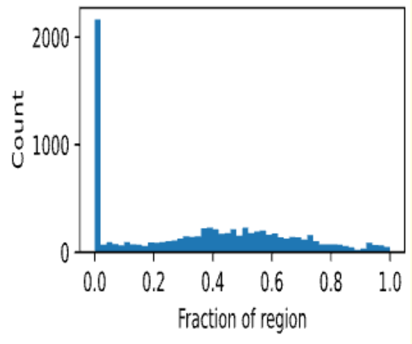	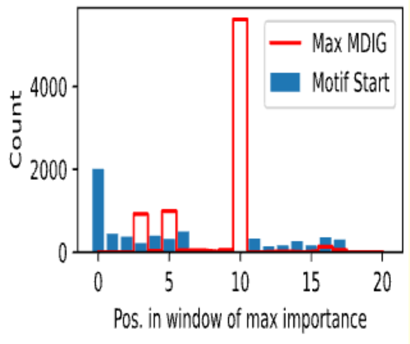	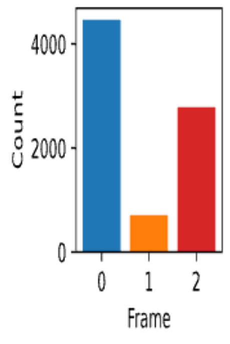	3.49E-07	1.40E-09	4.59
7	ORF	mRNAs (↑ NC)	mRNAs (random)	3455/26039 (13.3%)	1556/26039 (6.0%)	5	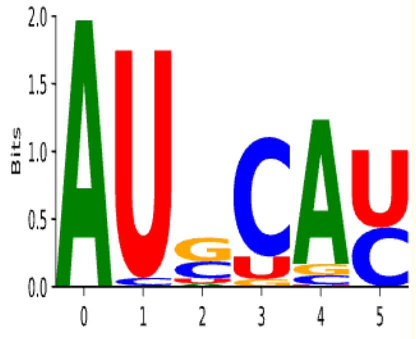	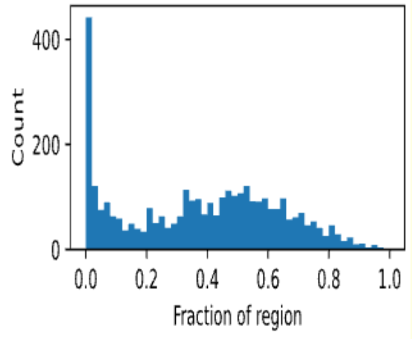	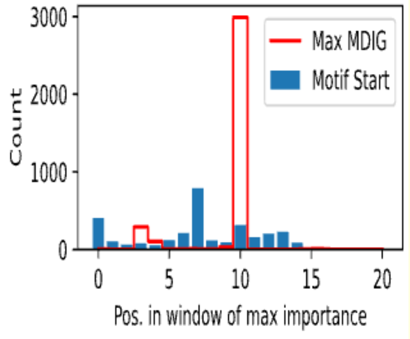	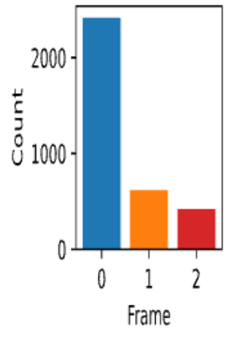	7.22E-10	2.90E-12	7.43

Motif #	Region	Positive Set (sites)	Negative Set (sites)	Pos. Sites	Neg. Sites	Cluster	Logo	Start site in region	Start site in window	Offset from ORF	E-value	p-value	Information
8	ORF	mRNAs (↑ NC)	mRNAs (random)	4119/26039 (15.8%)	2712/26039 (10.4%)	6	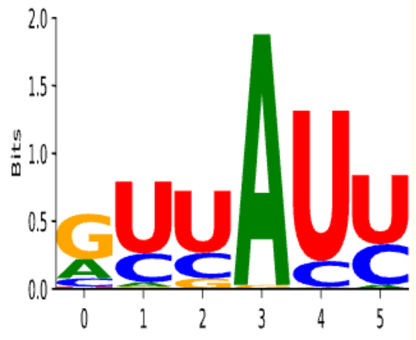	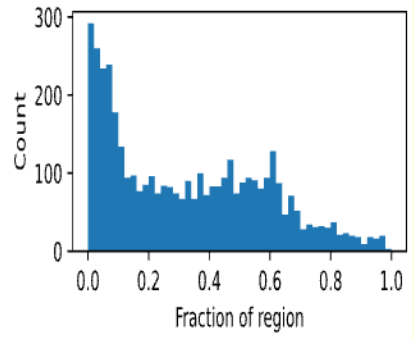	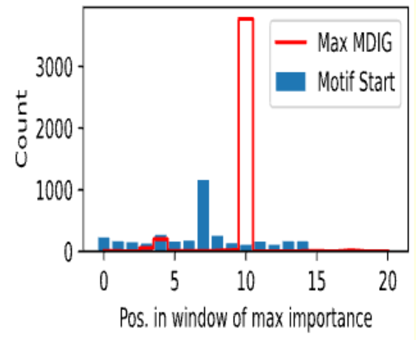	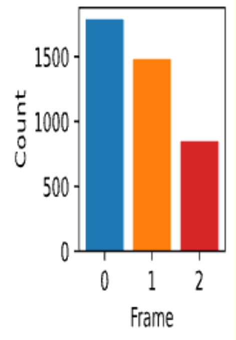	9.71E-06	3.90E-08	6.11
9	ORF	lncRNAs (↑ PC)	lncRNAs (random)	17756/24881 (71.4%)	13640/24881 (54.8%)	3	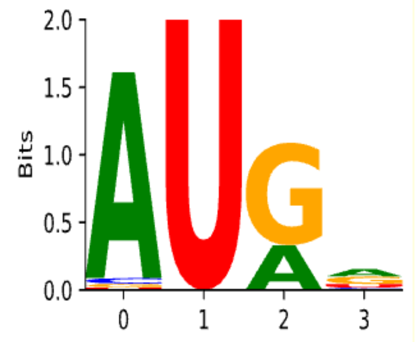	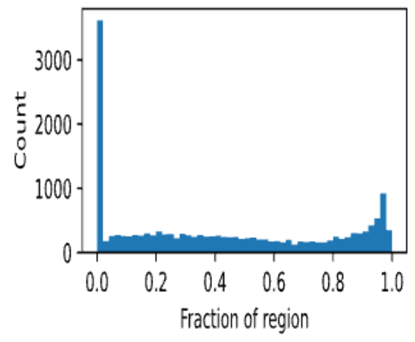	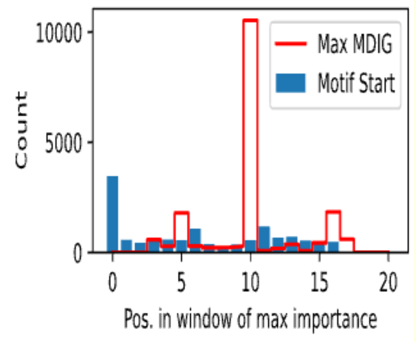	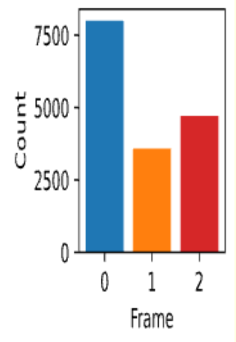	7.22E-28	2.90E-30	4.85
10	ORF	lncRNAs (↑ NC)	lncRNAs (random)	9939/24881 (39.9%)	5262/24881 (21.1%)	7	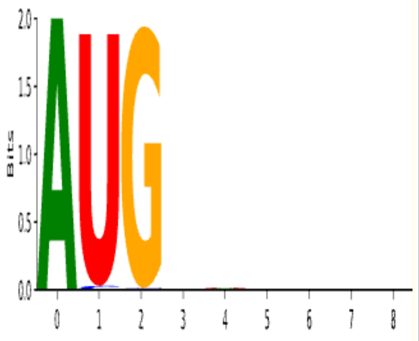	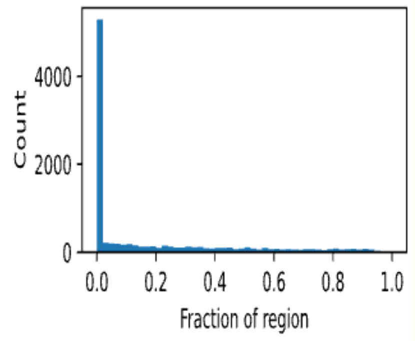	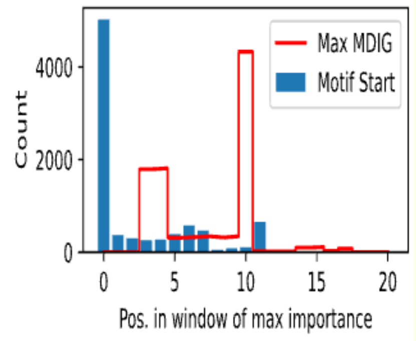	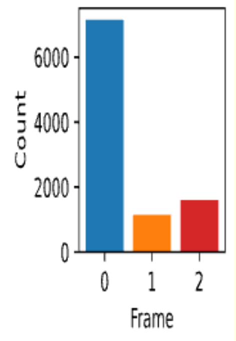	7.97E-52	3.20E-54	5.87
11	ORF	lncRNAs (↑ NC)	lncRNAs (random)	799/24881 (3.2%)	409/24881 (1.6%)	6	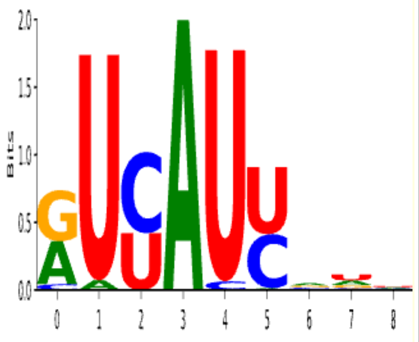	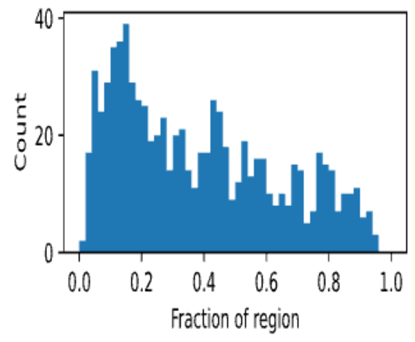	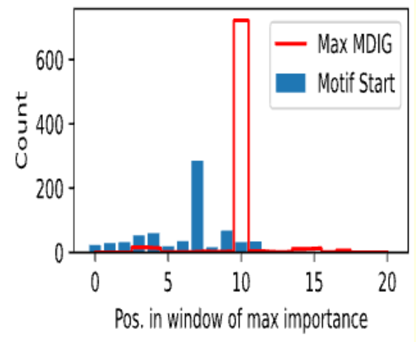	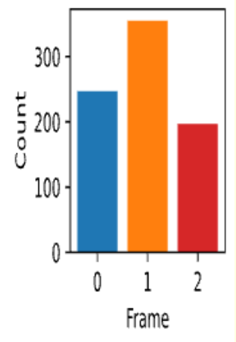	3.74E-04	1.50E-06	8.37

Motif #	Region	Positive Set (sites)	Negative Set (sites)	Pos. Sites	Neg. Sites	Cluster	Logo	Start site in region	Start site in window	Offset from ORF	E-value	p-value	Information
12	ORF	lncRNAs (↑ PC)	mRNAs (↑ PC)	2237/24881 (9.0%)	1181/26039 (4.5%)	8	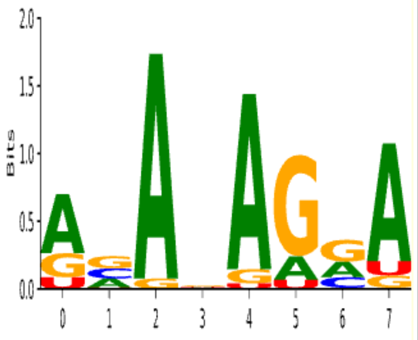	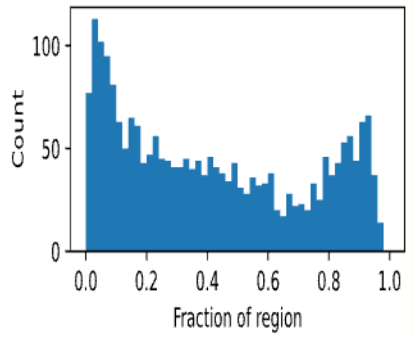	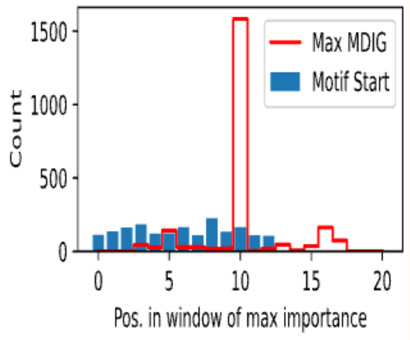	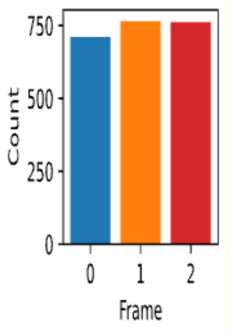	2.07E-05	8.30E-08	6.57
13	ORF	lncRNAs (↑ PC)	mRNAs (↑ PC)	3263/24881 (13.1%)	2356/26039 (9.0%)	9	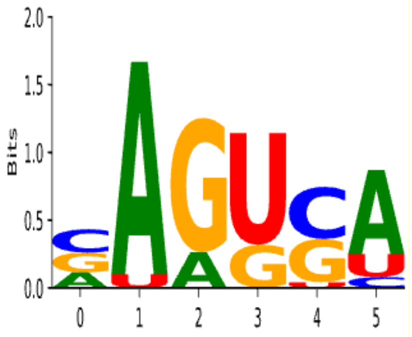	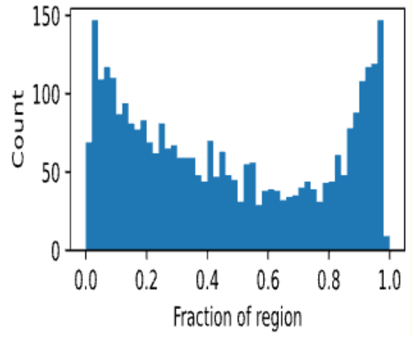	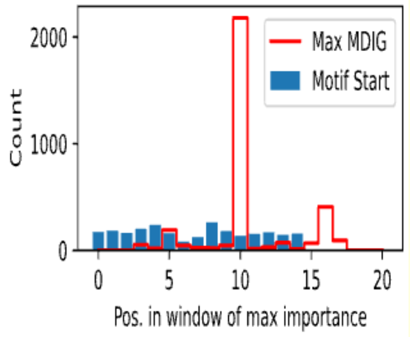	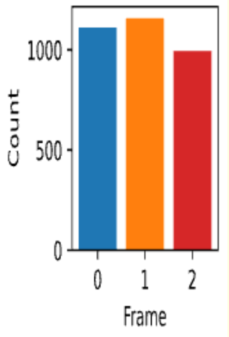	2.17E-04	8.70E-07	6.11
14	ORF	lncRNAs (↑ NC)	mRNAs (↑ NC)	3190/24881 (12.8%)	2315/26039 (8.9%)	8	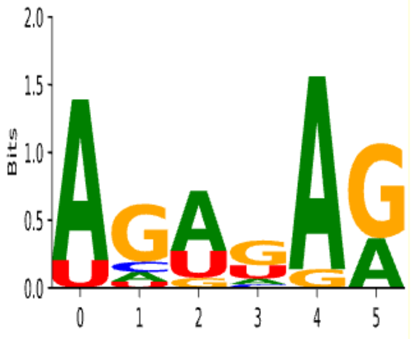	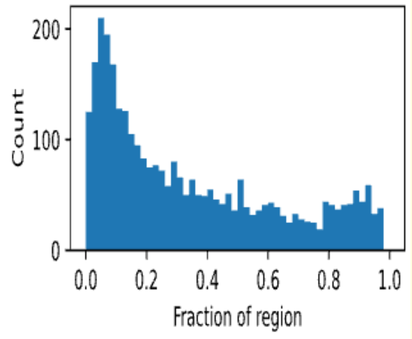	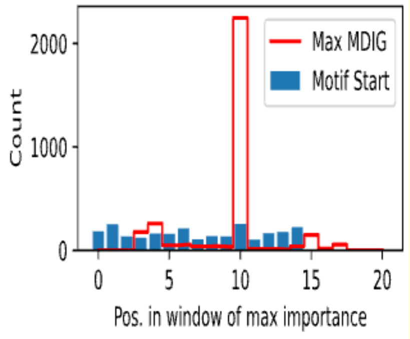	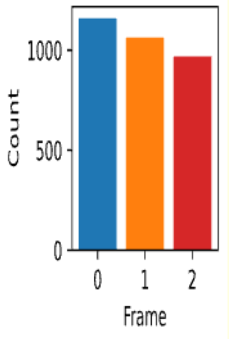	1.22E-07	4.90E-10	5.71
