# Supplementary material for: Improving deep models of protein-coding potential with a Fourier-transform architecture and machine translation task
Source: PLoS Comput Biol. 2023 Oct 12;19(10):e1011526. doi: 10.1371/journal.pcbi.1011526 (PMC10597526; doi:10.1371/journal.pcbi.1011526)
Supplement: Attachment — Submitted filename: Reviews_DH.pdf [file pcbi.1011526.s007.pdf]

We thank the reviewers for their time and for the overall constructive and positive reviews. We were pleased to see that Reviewer #1 stated that “the paper is well written and experiments are convincing” and that Reviewer #3 stated that using “FFT on this task is a novel idea” and that “this work represents a meaningful advance in the field, and the analyses were overall carefully performed”. We have centered our revision around three main critiques distilled from the reviews:

1. The translation task may be computationally excessive because a protein translation is deterministic given the location of its CDS. Related to this, the relative weighting of the classification and translation subtasks may be underexplored in our design of bioseq2seq.
2. The equivalence of FFT-based filtering and convolution suggests a comparison to convolutional neural network (CNN) models.
3. Our efforts to reduce sequence homology between the training and evaluation sets may have been insufficient.

We concur with the reviewers that locating the CDS is a crucial intermediate step in determining the protein translation, and have therefore trained a third model type to predict the index of the start codon for mRNAs. In addition, we have added an experiment exploring the role of differentially weighting the classification token and the subsequent amino acid tokens in the loss function for bioseq2seq. To empirically compare and contrast LFNet and convolutional architectures, we also train CNN models for all four training tasks. In total, we present results from five replicates for each of the eight combinations of neural architecture and training task. We hope this comprehensive study delineates the performance contributions of each design decision. Lastly, we clarify that our original protocol for minimizing test-set homology follows common practices, implement additional filtering methods, and demonstrate that model performance depends only modestly on the remaining sequence homology. Overall, we believe the major conclusions of our work remain intact and have been strengthened by the additions.

We additionally fixed an error in our definition of MDIG, with Eq. 4 in the prior submission missing a second term for the endogenous nucleotide character and some notational inconsistencies. No empirical results related to MDIG were affected by this mistake, only the text was incorrect. We regret the mis-transcription and have added additional exposition of the mathematical intuition behind MDIG to improve confidence in its correctness. Detailed point-by-point responses to reviewer comments follow below.

Reviewer #1: In this work, the authors augment a model to predict protein-coding potential with an additional task to output the sequence of the protein produced in case of protein-coding genes. The authors interpret the models using ISM and MDIG, a variant of Integrated Gradients developed by the authors to approximate ISM. They show that augmenting the model with the sequence translation task improves model interpretability. Overall, the paper is well written and experiments are convincing. I have some comments regarding the methods and suggestions for experiments.

Major:

- In my opinion, a more direct solution would be to predict only the start and end indices of the protein-coding transcript. That would provide the model the same information as predicting the protein sequence, as the mapping from (start,end) to sequence is deterministic. This would simplify to predicting the start and end indices auto-regressively, similar to methods employed for extracting subsequences for in the case of question-answering (see https://arxiv.org/pdf/1608.07905.pdf). Could the authors comment on their design choice? If possible, can the authors show that outputting the protein sequence performs better compared to simply outputting the start and end coordinates of the CDS.

>> We agree that identifying the CDS would require the model to learn much of the same information as predicting the protein sequence, apart from the genetic code. We have implemented another training task called bioseq2start which further simplifies the suggested task of predicting (start,end) to only predicting the start codon index. We take this to be the logical extension of the idea that we should train on the minimal task needed to inject information about the CDS into the learning process. We believe this simplification is justifiable given that stop codon readthrough and other instances of noncanonical termination are rare in eukaryotes, and therefore the protein sequence is deterministic given only its start codon. In addition, this removes the need for an autoregressive decoder, and we implement an attention-based index selection mechanism similar to one in the provided reference. We find that this setup does outperform the original bioseq2seq task but suffers from a lack of robustness in the form of extreme threshold sensitivity. This in turn hampered our interpretation efforts, where sensible log probabilities are desired. We suspect that this is an intrinsic difficulty in optimizing a categorical loss function over a large and variable number of categories. In addition, bioseq2start was ultimately surpassed by a re-weighted bioseq2seq inspired by your later comment.

- Can the authors also report metrics such as AUROC/AUPRC that would not depend on the specific threshold used for binary classification.

>> We have added both AUROC and AUPRC to Table 1. These threshold-independent metrics are the exception to the newly observed improvements over RNAsamba, likely because the dual role that the bioseq2seq decoder plays in performing classification and conditional sequence generation skews the calibration of its output distribution on the classification task. However, we feel strongly that this is outweighed by bioseq2seq's strong performance at the implicit (argmax) threshold and myriad benefits for interpretability. The loss function weighting technique also provides a natural way for users to achieve desired performance tradeoffs.

- Do the authors treat the relative weights of the two tasks (binary + sequence generation) as a hyper parameter? If not, why?

>> In bioseq2seq, the two tasks of binary and sequence generation are delineated only by position in the decoding, with the leading token playing the role of a classification token. In our original submission, we assigned equal weight to the (negative log likelihood) loss computed for

each output position. To explore your suggestion of differentially weighting the two tasks, we have added an experiment in which the overall loss function is reweighted using an instance-specific affine combination where weights decay exponentially with position with a new models called “bioseq2seq-weighted” (bioseq2seq-wt). Doing this leads to a model with better classification performance than the uniform bioseq2seq task. We think this provides further support to the idea that translation improves the classification task, but also shows that a minimal component of translation is sufficient to get a large boost in performance.

- The authors mention that "when bioseq2seq was allowed to predict a full-length protein rather than...the classification performance of the best model deteriorated slightly...": I am not sure why this is the case. My understanding is that the binary prediction is separate from the protein prediction (indeed it is modelled as such), and the predicted protein sequence is conditioned on the case that the prediction is protein-coding. The accuracy of the binary task should not be affected by the sequence generation.

>> The reduced binary classification performance when protein sequences are generated is due to the necessity of using heuristic decoding strategies at inference time. During beam search, the score for each hypothesis sequence (beam) is calculated by summing the log probabilities for each output token and dividing by the length of the hypothesis. This length-normalization heuristic is carried over from natural language processing to adjust for bias against longer sequences. However, it can improperly reward longer decodings, which under our formulation must be protein-coding because <NC> is always of length 1. Thus, while your observation that “binary prediction is separate from the protein prediction” is correct, subsequent steps of beam-search may lead to a <PC> hypothesis receiving the highest overall score even where the initial classification token probabilities would imply a prediction of <NC>. We have added language to clarify how we treat the class prediction when a full prediction is made.

- Many of the questions and hypotheses posed in the discussion can be explicitly tested using in-silico experiments:

= the authors can test how the prediction changes as the length of the CDS is reduced by changing the position of the start and/or stop codons

= the authors can test how the presence/absence of upstream ORFs (their length, relative position) is predicted to affect coding potential

>> We have added an additional experiment exploring the effect of uORFs on coding potential by swapping the endogenous 5’ UTRs of mRNAs lacking a uORFs with 5’ UTRs from other mRNAs which do harbor uORFs. The idea here was to guarantee the use of natural 5’ UTRs while specifically intervening on the presence of a uORF. These results are presented both in the main text and Supplementary Fig 6. To get at the effect of CDS length, we expanded our analysis

which originally focused on introducing stop codons within 50 codons of the start, and extended it to include nonsense mutations along the whole length of CDS bins, as shown in Figure 4C.

- The authors may choose to attempt to answer some other interesting questions using in-silico experiments: in how many edits can a coding transcript be converted to non-coding (without breaking start codons or creating stop codons). The converse (lncRNA->mRNA) conversion can also be tried.

>>Thank you for this suggestion. We agree that this would be a great follow-up experiment, but we feel it is too much to include with the current manuscript because some preliminary experiments suggest this would involve a lot of computational follow-up and would be best presented alongside experimental validation of the predictions.

Minor:
- Specify full form of MCC in Table 1

This has been added under “Evaluation Metrics”.

- Table 2 is not cited in the main text

>>Thanks for catching this, we have fixed this.

- typo "as an transcript-level" (Page 10)

>>This has also been fixed.

- The masking strategy for interpretation bears some resemblance to https://pubmed.ncbi.nlm.nih.gov/35966405/, and the authors may choose to explore the interpretation strategies outlined in the paper

We find the provided reference interesting but in light of the significantly expanded scope of the paper required to explore multiple architectural and training task variants, we feel introducing a third interpretive strategy beyond ISM and MDIG could cloud the exposition of the paper.

- more potential explanation on why UGG may increase non-coding potential would be useful
>>

Our new best model using the bioseq2seq-weighted training task did not appear to emphasize UGGs in particular, which moots this particular observation.

Reviewer #2: In this manuscript, the authors proposed bioseq2seq, a seq2seq formulation for predicting protein-coding potential with deep neural networks and demonstrate that simultaneously learning translation from RNA to protein improves classification performance relative to a classification-only training objective. Bioseq2seq introduces Local Filter Network (LFNet), a computationally efficient network layer based on the short-time Fourier transform. LFNet is a network architecture with an inductive bias for modeling the three-nucleotide periodicity apparent in coding sequences. the authors incorporate LFNet within an encoder-decoder framework to test whether the translation task improves the classification of transcripts and the interpretation of their sequence features. The resulting model is then used to compute nucleotide-resolution importance scores, revealing sequence patterns that could assist the cellular machinery in distinguishing mRNAs and lncRNAs.

See the detailed comments below:
1. The output of bioseq2seq is either for protein coding and for noncoding followed by the translated protein in the case of and nothing in the case of . However, bioseq2seq's formulation seems unnecessary. In principle, if the status (i.e., or) is predicted, the sequence inside (i.e., the translated protein or nothing) is deterministic. That said, simple binary classification seems sufficient and seq2seq is overkill.

>>Thank you for this point. We agree that comparison to binary classification is important, and for this reason we compared to what was previously called Encoder-Decoder Classifier (EDC) model in our manuscript, which has all the network architecture of bioseq2seq, but is only a classification algorithm. To clarify that this was one of our models, we have renamed EDC to bioseq2class. One of the core findings of our manuscript is that learning the translation task, and going beyond binary classification, helps the neural network learn mRNAs vs lncRNAs. Furthermore, we show that bioseq2seq is more interpretable because it learns the translation task.

That said, we also could interpret your comment similarly to Review 1's comment about prediction of the start/end indices of the coding sequence, from which the translation is deterministic. To address this, we have added several new models, including bioseq2start, which outputs only the position of the start codon, and weighted bioseq2seq, which is an intermediate between bioseq2seq and bioseq2class. All of the trained tasks involving some level of supervision from the translation product significantly outperform the direct classification setup in distinguishing mRNAs and lncRNAs. This illustrates that training a model in this fashion is worthwhile even if predicting translated proteins is not taken to be useful in and of itself. In this sense, *inference*-time translation capability is secondary to the impact of the seq2seq (or seq2start) formulation on the *training* procedure.

2. According to Fig 2, the predicted proteins deviate from the true protein in the status , suggesting that the seq2seq model is not as reliable as translating the protein using the deterministic rule.

>> This is true. Heuristic decoding strategies and our initial choice not to differentially weight different output positions mean that predicted translations can be imperfect. During inference, as you note, the deterministic rule can be used once <PC> has been predicted. In our new results on bioseq2seq-weighted, we observe that even poorer predictions at the character level can still distinguish the proper open reading frame with high accuracy. For some applications such as micropeptide discovery, reliable translation may be desirable to establish the cryptic ORF being translated. Even here, however, highly imperfect protein predictions can suffice to unambiguously establish the putative translated ORF using the deterministic rule.

3. The classification performance in Table 1 is not very convincing because the baseline methods are not comprehensive and relatively weak. That said, the major claim of the paper "simultaneously learning translation from RNA to protein improves classification performance relative to a classification-only training objective" cannot be well supported. More comprehensive evaluation is needed.

>>We agree on the importance of supporting the core hypothesis of the paper in Table 1. For that reason, we have included the state-of-the-art method, RNAsamba, as a comparison. Additionally, we have included CPAT and CPC2, because these methods are widely used in genomics, and widely used as a benchmark of performance for the transcript classification task. These methods provide a range of performance in which to test our core hypothesis. In addition to supporting our hypothesis with these methods commonly used in the field, we have tested our hypothesis by comparing to different training strategies using similar architectures to get at this core hypothesis. We compared bioseq2seq to the encoder-decoder classifier (EDC) because these two approaches differ only by the inclusion of the protein sequence prediction (translation) task. Furthermore, in the current revised manuscript, we have added an additional training strategy whereby we train a similar network to predict the location of the start codon, as a training strategy for the classification task. Under this approach the network predicts the position of the last nucleotide when the transcript is deemed to be noncoding. In summary, we are comparing the network that learns translation to improve classification performance (bioseq2seq) to both state-of-the-art and widely used tools, as well as similar architectures but without translation as a way of testing the effect of training strategy.

4. The core feature of LFNet is its learned frequency-domain filters whose frequency bin equivalent to a period of 3 nt. Though it sounds novel, a trivial alternative can be using CNN with filter kernel size=3 and stride=3. Justification is needed why the proposed frequency-domain filters are better. Maybe an ablation study is needed

>> One clarification is that LFNet does not exclusively process the 3nt frequency signal. In light of the 3nt periodicity property of coding frequencies, we hypothesized that it would learn a strong emphasis on this component, but the filter matrix multiplies all bands possible given the

“sampling frequency” of 1 nt. The proposed alternative would seem to only anticipate the 3nt band. However, we do acknowledge a strong connection between our method and CNNs via the convolution theorem of Fourier Transforms. LFNet essentially implements large-kernel convolutions in the frequency domain rather than the time domain. For this reason, we have comprehensively compared LFNet in all four of our training tasks to separately optimized CNNs, allowing for exponentially increasing dilation factors to expand the receptive field of the network. Based on these results, we cannot claim that LFNet systematically outperforms more conventional CNNs, but it does appear mildly better and more stable in the overall best-performing bioseq2seq-weighted setting as well as in several models for the lncPEP scoring experiment.

Reviewer #3: In this manuscript, the authors developed a new approach for protein-coding potential prediction from RNA sequences using a FFT-based frequency domain neural network architecture and a machine translation task. Using FFT on this task is a novel idea that utilizes the 3nt periodicity of the protein-coding sequence. The models proposed by the authors achieve comparable performance with prior art such as RNAsamba without relying on pre-selected input features. In addition, the authors performed extensive analysis to interpret the model, including testing a new approach of using an integrated gradient method to approximate the predicted mutation effect from saturated in silico mutagenesis (ISM). Overall, this work represents a meaningful advance in the field, and the analyses were overall carefully performed. My detailed comments are below:

Major points:

1. In this study, the authors collected sequences from eight mammalian species, which were divided into training, validation, and test sets. Based on my understanding of the method section, even though the authors made an effort to reduce the similarity between training and test set by excluding those with >80% identity, the test set may still contain sequences with strong homology to the training set sequences. Can the authors make sure that the test set sequences do not liftover to training set sequences? Can the test set be stratified by maximal similarity to the training set and compare performance across different levels of similarities?

>> We thank you for flagging this concern. We ran CD-HIT with settings suggested by its documentation and in line with prior work, including CPPred, which even lists the same command line options as we used. However, while investigating your concerns, we found that around 25% of test sequences considered by CD-HIT to be below the 80% similarity threshold with all training examples exceed this threshold when double-checked with full Needleman-Wunsch alignment seeded by significant BLAST hits. These seemingly “missed” similar sequences can be explained by several CD-HIT heuristics including banded alignment and short word filtering to avoid making exhaustive pairwise alignments between all examples. To address this discrepancy, we have further reduced our test set to remove the sequences that match

either the training set or the validation set according to this secondary alignment pipeline. The results in the manuscript now address only this reduced dataset. In addition, we have added Supplementary Figure 2 depicting the relationship between classification performance and maximal similarity to the training set for all methods. This reveals a slight dependence on similarity for all methods, but good performance even for distant sequences.

2. For translation tasks, what happens when the produced translation does not exactly match the ground truth? It seems like a task that should achieve 100% accuracy if the translation start site is correct.

One piece of evidence comes from evaluating seq2seq models on the validation data during training. For parallel efficiency the conditioning of downstream predictions is driven using a masked version of the (validation) ground truth sequence rather than actual autoregressive model predictions. This allows us to compute a full position-wise accuracy, which typically exceeds 99.5% for the unweighted bioseq2seq models, suggesting strongly that the rote application of the genetic code can be done very accurately in principle. At test-time however, we must use beam search to perform autoregressive prediction. During beam search, the score for each hypothesis sequence (beam) is calculated by summing the log probabilities for each output token and dividing by the length of the hypothesis. This length-normalization heuristic is carried over from natural language processing to adjust for bias against longer sequences. It can however lead to unpredictable behavior with respect to translation accuracy. Uncertainty or errors on to the leading amino acids of a protein can cause the prediction to drift towards open alternate reading frames or even hallucinate spurious output.

Minor points:
1. The Fourier transform has a direct relationship to convolution, as convolution can be implemented with the FFT transform. It would be worthwhile to discuss the differences and advantages of this particular approach compared to the traditional convolution approach, considering this connection.

>> We agree that by the convolutional theorem of the Fourier Transform, the LFNet elementwise multiplication on a frequency-domain representation implements a convolutional algorithm in the sequence domain. We have added discussion pointing out this connection as well as the fact that LFNet permits large kernel convolutions. Based on the interest of several reviewers in the link between LFNet and convolution, we have added a comprehensive comparison to more conventional CNN approaches. Based on these results, we cannot claim that LFNet systematically outperforms more conventional CNNs, but it does appear mildly better and more stable in the overall best-performing bioseq2seq-weighted setting as well as in several models for the lncPEP scoring experiment.

2. How much performance improvement does Softshrink in the authors' architecture provide, which is an unusual and interesting design, compared to using a regular activation function after IFFT?

>>In choosing to apply Softshrink after the weight matrix, we followed the Adaptive Fourier Neural Operator paper. Softshrink is motivated as the proximal operator for the L1 norm, which serves to induce sparsity in the learned weight matrix. In practice, we do not find that this creates noticeable amounts of sparsity, and across the four training tasks, we observe that hyperparameter tuning selected a wide range of threshold values, implying that the contribution to performance is likely minimal. We would also like to clarify that this is not the only activation function used, as LFNet includes a Transformer-style feed forward network containing ReLU activations.

3. Does the inductive bias favor the utilization of protein-coding regions over UTRs? Is it as effective as convolution-based approaches in detecting translation start signals? The model appears to capture the downstream 2bp of the Kozak sequence. What about the Kozak sequence upstream of ATG?

>> Three-nucleotide periodicity is widely taken to be a consequence of unequal codon usage in CDS regions, so to the extent that this property is weaker or nonexistent in 5' and 3' UTRs, the LFNet inductive bias will serve to distinguish the three regions. When slicing sequence contexts around importance maxima for motif discovery, we prevented the chosen slice from spanning two functional regions, e.g. the 5' UTR and CDS, which the Kozak sequence does. Thus, the motif discovery protocol is biased against highlighting such a feature.

4. Can the performance of the pretrained model RNAsamba be compare to the model proposed by authors?

>> Because we designed our own train/test/validation data and redundancy reduction protocol, the pretrained version of RNAsamba will have been trained on examples from our testing set, making it unsuitable to compare to other models under best evaluation practices. Even with this significant caveat, we see that the pretrained version called 'partial_length_weights.hdf5' obtains an F1 of 0.917 and and the one called 'full_length_weights.hdf5' scores 0.926 below our best models and the RNAsamba that we trained ourselves.
